# Metabolomics combined with intestinal microbiota reveals the mechanism of compound Qilian tablets against diabetic retinopathy

**DOI:** 10.3389/fmicb.2024.1453436

**Published:** 2024-08-16

**Authors:** Jiangwei Jia, Bo Liu, Xin Wang, Fenglan Ji, Fuchun Wen, Huibo Xu, Tao Ding

**Affiliations:** Pharmacodynamic and Toxicological Evaluation Center, Jilin Academy of Chinese Medicine Sciences, Changchun, China

**Keywords:** diabetic retinopathy, compound Qilian tablets, metabolomics, intestinal microbiota, metabolic pathway

## Abstract

**Background:**

Diabetic retinopathy (DR) is one of the common chronic complications of diabetes mellitus, which has developed into the leading cause of irreversible visual impairment in adults worldwide. Compound Qilian tablets (CQLT) is a traditional Chinese medicine (TCM) developed for treating DR, but its mechanism is still unclear. This study explored the mechanism of action of CQLT in treating DR through metabolomics and intestinal microbiota.

**Methods:**

Histopathologic examination of the pancreas and retina of Zucker diabetic fatty (ZDF) rats and immunohistochemistry were used to determine the expression levels of retinal nerve damage indicators ionized calcium binding adaptor molecule-1 (Iba-1) and glial fibrillary acidic protein (GFAP). Rat fecal samples were tested by LC-MS metabolomics to search for potential biomarkers and metabolic pathways for CQLT treatment of DR. Characteristic nucleic acid sequences of rat intestinal microbiota from each group were revealed using 16S rDNA technology to explore key microbes and related pathways for CQLT treatment of DR. At the same time, we investigated the effect of CQLT on the gluconeogenic pathway.

**Results:**

After CQLT intervention, islet cell status was improved, Iba-1 and GFAP expression were significantly decreased, and abnormal retinal microvascular proliferation and exudation were ameliorated. Metabolomics results showed that CQLT reversed 20 differential metabolites that were abnormally altered in DR rats. Intestinal microbiota analysis showed that treatment with CQLT improved the abundance and diversity of intestinal flora. Functional annotation of metabolites and intestinal flora revealed that glycolysis/gluconeogenesis, alanine, aspartate and glutamate metabolism, starch and sucrose metabolism were the main pathways for CQLT in treating DR. According to the results of correlation analysis, there were significant correlations between Iba-1, GFAP, and intestinal microbiota and metabolites affected by CQLT. In addition, we found that CQLT effectively inhibited the gluconeogenesis process in diabetic mice.

**Conclusion:**

In conclusion, CQLT could potentially reshape intestinal microbiota composition and regulate metabolite profiles to protect retinal morphology and function, thereby ameliorating the progression of DR.

## 1 Introduction

As a metabolic disease that seriously threatens human health, diabetes is one of the fastest-growing global health emergencies in the 21st century and has become a common disease (Fan et al., 2022; Kerper et al., 2022). About 537 million adults between the ages of 20 and 79 have diabetes worldwide, which will surge to 783 million by 2045, according to the International Diabetes Federation (Yuan et al., 2024). Diabetic retinopathy (DR) is the leading cause of vision loss in working-age people worldwide. Among people with diabetes, the overall prevalence of DR is 22.27%, and the number of adults with DR globally is estimated to be 103.12 million in 2020, and by 2045 this number is projected to increase to 160.5 million (Teo et al., 2021). With a history of diabetes for more than 15 years, almost all people with type 1 diabetes and more than 60% of people with type 2 diabetes develop retinopathy (Klein et al., 2008). DR has complex pathogenesis, inflammatory response, oxidative stress, retinal ischemia and hypoxia, autophagy, retinal neurodegeneration, and abnormal retinal microvascular proliferation are widely recognized as its coexisting pathogenesis, which leads to the challenging treatment of DR (Semeraro et al., 2015; Aouiss et al., 2019; Wang et al., 2022). Currently, the main treatments for DR include retinal photocoagulation, vitrectomy, or anti-VEGF therapy; however, surgical treatments may not only lead to retinal damage and scarring but also bring economic burdens to patients (Dervenis et al., 2023; Lin et al., 2023). Patients with DR have nearly double the healthcare costs compared to those without DR (Heintz et al., 2010). Therefore, prevention and treatment of DR are of great significance to diabetic patients and the entire society. Traditional Chinese medicine (TCM) treatment of DR often uses herb and their formulas that have the effects of clearing heat and fire, activating blood circulation and removing blood stasis, and tonifying Qi; they have the advantages of precise efficacy, low price, fewer side effects, and easy accessibility (Zhang et al., 2018; Ai et al., 2020). Compound Qilian tablets (CQLT) were developed based on the clinical experience and theories of TCM and consist of four herbs, including *Astragalus membranaceus*, *Rehmannia glutinosa*, *Coptis chinensis*, and *Panax notoginseng*, and are used for blurred vision, visual field loss, and vitreous hemorrhage caused by DR. We have previously found that CQLT exerts anti-DR effects through multi-components, multi-targets, and multi-pathways, which coincides with the complex pathogenesis of DR and highlights the characteristics of CQLT for treating DR (Jia et al., 2024).

Increasing evidence shows that TCM benefits disease by regulating metabolites and intestinal flora. Therefore, it is necessary to investigate the relationship between the anti-DR effect of CQLT and intestinal flora and metabolites (Che et al., 2022; Li et al., 2023). Metabolomics is dynamic, holistic, and systematic as an essential part of systems biology. It can more intuitively and effectively understand biological processes and mechanisms. The biomarkers it provides have great potential in diagnosing and treating diseases (Johnson et al., 2016; Rinschen et al., 2019). Metabolites, as downstream genomics products, are closest to the disease phenotype. Searching for biomarkers of DR by detecting specific metabolites in DR patients is of great significance for the treatment and diagnosis of DR (Roberts et al., 2012). The application of metabolomics can identify metabolite disorders in DR and analyze the biomarkers and related metabolic pathways of CQLT in treating DR. Intestinal flora assumes some essential functions in the body’s immunity, metabolism, structure, and nervous system and plays an indispensable role in maintaining normal physiological functions of the human body (Leclercq et al., 2016). Disruption of its homeostasis has been correlated with various diseases, such as inflammatory bowel disease, diabetes, and cardiovascular disease (de Vos et al., 2022; Guo et al., 2022). Meanwhile, an increasing body of research indicates that the gut microbiota is closely related to the host’s ocular health and plays a significant role in conditions such as glaucoma, DR, and age-related macular degeneration (Napolitano et al., 2021; Serban et al., 2023).

We use Zucker diabetic fatty (ZDF) rats to construct DR models for research. ZDF (fa/fa) rats are spontaneous diabetes and obesity animal models that can effectively simulate the pathogenesis of human DR. ZDF (fa/+) rats are ZDF (fa/fa) rat syngeneic standard control (Wohlfart et al., 2014; Szabó et al., 2017). In this study, we used metabolomics to study the fecal metabolites of ZDF rats and investigate the characteristic metabolites and metabolic pathways of CQLT to treat DR. The 16S rDNA technique revealed the dominant intestinal flora in each experimental group and explored the differential microorganisms and associated pathways in DR treated with CQLT. In addition, relevant metabolic pathways were experimentally verified to study further the mechanism of action of CQLT in treating DR.

## 2 Materials and methods

### 2.1 Materials

CQLT (Lot: 210305T) was produced by Jilin Yatai Yongantang Pharmaceutical Co., Ltd. (Changchun, China). It was prepared by combining *Astragalus membranaceus*, *Rehmannia glutinosa*, *Coptis chinensis*, and *Panax notoginseng* through the corresponding process to form film-coated tablets. The primary antibodies ionized calcium binding adaptor molecule-1 (Iba-1, DF6442) and glial fibrillary acidic protein (GFAP, DF6040) were obtained from Affinity Biosciences (Jiangsu, China). The secondary antibody HRP-conjugated Affinipure Goat Anti-Rabbit IgG (H + L) (SA00001-2) was purchased from Proteintech (Wuhan, China). Streptozotocin (STZ, Lot: WXBC8740V) was purchased from Sigma-Aldrich (Shanghai, China); l-alanine (Lot: 1BB10124) was purchased from Beijing Dingguo Changsheng Biotechnology Co., Ltd. (Beijing, China).

### 2.2 Animal experiments

We purchased Specific Pathogen Free (SPF) grade 8–9 weeks male ZDF (fa/fa) and ZDF (fa/+) rats, weighing 280–320 g, from Beijing Vital River Laboratory Animal Technology Co., Ltd. (Beijing, China). SPF grade *K5008* specially processed feed was purchased from Beijing Keao Xieli Feed Co., Ltd. (Beijing, China, Lot No. 2022092808); SPF grade rat and mouse maintenance feed was purchased from Liaoning Changsheng Biotechnology Co., Ltd. (Liaoning, China, Lot No. 22081211). ZDF (fa/fa) rats were fed with *K5008* feed, and ZDF (fa/+) rats (control group, *n* = 12) were fed with standard maintenance feed, and all animals were free to eat and drink. After 3 weeks of acclimatization, ZDF (fa/fa) rats with fasting blood glucose >16.7 mmol/L were randomly divided into a model group (*n* = 17) and a CQLT group (*n* = 13), and the CQLT group was given CQLT 0.337 g/kg once daily for 8 weeks. In the 6th week of administration, one rat in the control group and two in the model group were randomly selected for pathological retina examination. During the experiment, 3 and 1 rats died naturally in the model and CQLT groups, respectively. At the end of CQLT treatment, rats were placed in metabolic cages to collect feces from rats in the control (*n* = 11), model (*n* = 12), and CQLT groups (*n* = 12), and the feces were placed in liquid nitrogen for 24 h. Afterward, these 35 fecal samples were stored at −80°C for metabolomics and intestinal microbiota analysis.

Fifty SPF male Institute of Cancer Research (ICR) mice weighing 20–24 g were purchased from Changchun Yisi Experimental Animal Technology Co., Ltd. (Changchun, China). After adapting to the laboratory for 3 days, they were divided into a control group (*n* = 10) and a modeling group (*n* = 40) according to the principle of blood glucose balance. After grouping and fasting for 12–16 h, mice in the modeling group were injected intraperitoneally with STZ 150 mg/kg. Random blood glucose was measured 5–7 days after STZ injection, and animals with blood glucose less than 11.1 mmol/L were eliminated. The animals were divided into the model group (*n* = 9), metformin group (0.4 g/kg, *n* = 10), and CQLT group (1.38 g/kg, *n* = 11) according to the principle of blood glucose balance. Four days after the administration of the drug, the animals fasted for 12 h, the corresponding drug solution was given, and blood glucose was measured 1 h after the administration of the drug; l-alanine (2 g/kg) was injected intraperitoneally, and blood glucose was measured again 60 min after the injection, and the ability of gluconeogenesis was evaluated by the blood glucose rise value 60 min after the injection of l-alanine. The animals were housed in the Barrier Environment Animal Laboratory of Jilin Academy of Chinese Medicine Sciences (Changchun, China) under controlled conditions of 20–25°C temperature, 50–60% relative humidity, and a 12 h light/dark cycle. All animal experiments were reviewed and approved by the Animal Ethics Committee of Jilin Academy of Chinese Medicine Sciences (No. JLSZKYDWLL2018-003).

### 2.3 Pathological examination of pancreas and retina in ZDF rats

The rats were anesthetized after the last administration, and the pancreas and eyeballs were quickly removed and fixed in formalin solution for more than 48 h. The samples were routinely washed, dehydrated, embedded in paraffin wax, sectioned, and stained with hematoxylin–eosin (H&E). The pathological changes were observed under a microscope (Olympus Corporation, Japan).

### 2.4 Immunohistochemical analysis of retinal GFAP and Iba-1 in ZDF rats

Immunohistochemical methods were used to detect the expression of GFAP and Iba-1 in the retina of each group of rats. Detailed experimental methods are provided in Supplementary material S1. Finally, the average optical density values of the chromogenic positive substances were observed and measured under an optical microscope.

### 2.5 Untargeted metabolomics analysis

Four hundred microliters of solution (methanol: water = 7:3, V/V) containing the internal standard was added to 20 mg of the ZDF rat feces and vortexed for 3 min. After the sample was sonicated, vortexed, and centrifuged, 200 μL of the supernatant was taken for LC-MS analysis. All samples were for two LC/MS methods. One aliquot was analyzed using positive ion conditions and was eluted from the T3 column (Waters ACQUITY Premier HSS T3 Column 1.8 μm, 2.1 mm × 100 mm) using 0.1% formic acid in water as solvent A and 0.1% formic acid in acetonitrile as solvent B in the following gradient: 5 to 20% in 2 min, increased to 60% in the following 3 min, increased to 99% in 1 min and held for 1.5 min, then come back to 5% mobile phase B within 0.1 min, held for 2.4 min. The analytical conditions were as follows: column temperature, 40°C; flow rate, 0.4 mL/min; injection volume, 4 μL. Another aliquot used negative ion conditions, which was the same as the elution gradient of the positive mode. The data acquisition was operated using the information-dependent acquisition (IDA) mode using Analyst TF 1.7.1 Software (Sciex, Concord, ON, Canada). The source parameters were set as follows: ion source gas 1 (GAS1), 50 psi; ion source gas 2 (GAS2), 50 psi; curtain gas (CUR), 25 psi; temperature (TEM), 550°C; declustering potential (DP), 60 V, or −60 V in positive or negative modes, respectively; and ion spray voltage floating (ISVF), 5,000 V or −4,000 V in positive or negative modes, respectively. The TOF MS scan parameters were set: mass range, 50–1,000 Da; accumulation time, 200 ms. Sample quality control analyses were performed on the data structure, and multivariate statistical analyses such as grouped principal component analysis (PCA) and orthogonal partial least squares discriminant analysis (OPLS-DA) were performed on the standardized processed data. Potential differential metabolites were screened based on variable importance in the projection (VIP) >1, *p* < 0. 05, endogenous differential metabolites were identified through the human metabolome database,^1^ and the obtained differential metabolites were imported into MetaboAnalyst 6.0^2^ for metabolic pathway analysis (Chen et al., 2021).

### 2.6 16S rDNA analysis

DNA extraction from the ZDF rat feces was performed according to the instructions of the Fecal Genomic DNA Extraction Kit; after determining the DNA concentration and purity, the samples were stored at −80°C for use. 16S rDNA genes of distinct regions (16SV4/16SV3/16SV3–V4/16SV4–V5) were amplified used specific primer with the barcode. All PCR reactions were carried out with 15 μL of Phusion^®^ High-Fidelity PCR Master Mix (New England Biolabs), 0.2 μM of forward and reverse primers, and about 10 ng template DNA. Thermal cycling consisted of initial denaturation at 98°C for 1 min, followed by 30 cycles of denaturation at 98°C for 10 s, annealing at 50°C for 30 s, and elongation at 72°C for 30 s and 72°C for 5 min. The PCR products were mixed and purified for library construction; after the libraries were qualified by Qubit and Q-PCR quantification, PE250 sequencing was performed using NovaSeq6000. After getting raw tags, splice and filter to get clean tags. Tags sequences were aligned with the species annotation database to detect chimeric sequences and removed to obtain effective tags. Noise reduction of effective tags was performed using QIIME2 software to obtain amplicon sequence variants (ASVs) and feature sequences. Species annotation was performed using QIIME2 software, and species abundance was counted. The abundance and diversity of intestinal flora within the samples were reflected by alpha diversity (Li et al., 2013). Comparative analysis of microbial community composition of different samples using beta diversity (Huang et al., 2023). Functionally annotated information on gut microbiota was obtained using the Tax4Fun V0.3.1 R package (Yu et al., 2022).

### 2.7 Correlation analysis

Correlation analysis of retinal nerve injury indicators, significantly different gut microbiota genera, and metabolites was performed using the Spearman correlation analysis method to reveal the interactions between retinal nerve injury indicators, intestinal flora, and metabolites.

### 2.8 Data analysis

GraphPad Prism 9.5 statistical software was used to analyze the experimental data, and comparisons between the two groups were made by *t*-test, with *p* < 0.05 indicating significant differences. The experimental data were expressed as mean ± SEM.

## 3 Results

### 3.1 CQLT ameliorated retinal and pancreatic injury in DR rats

**Figure 1:**
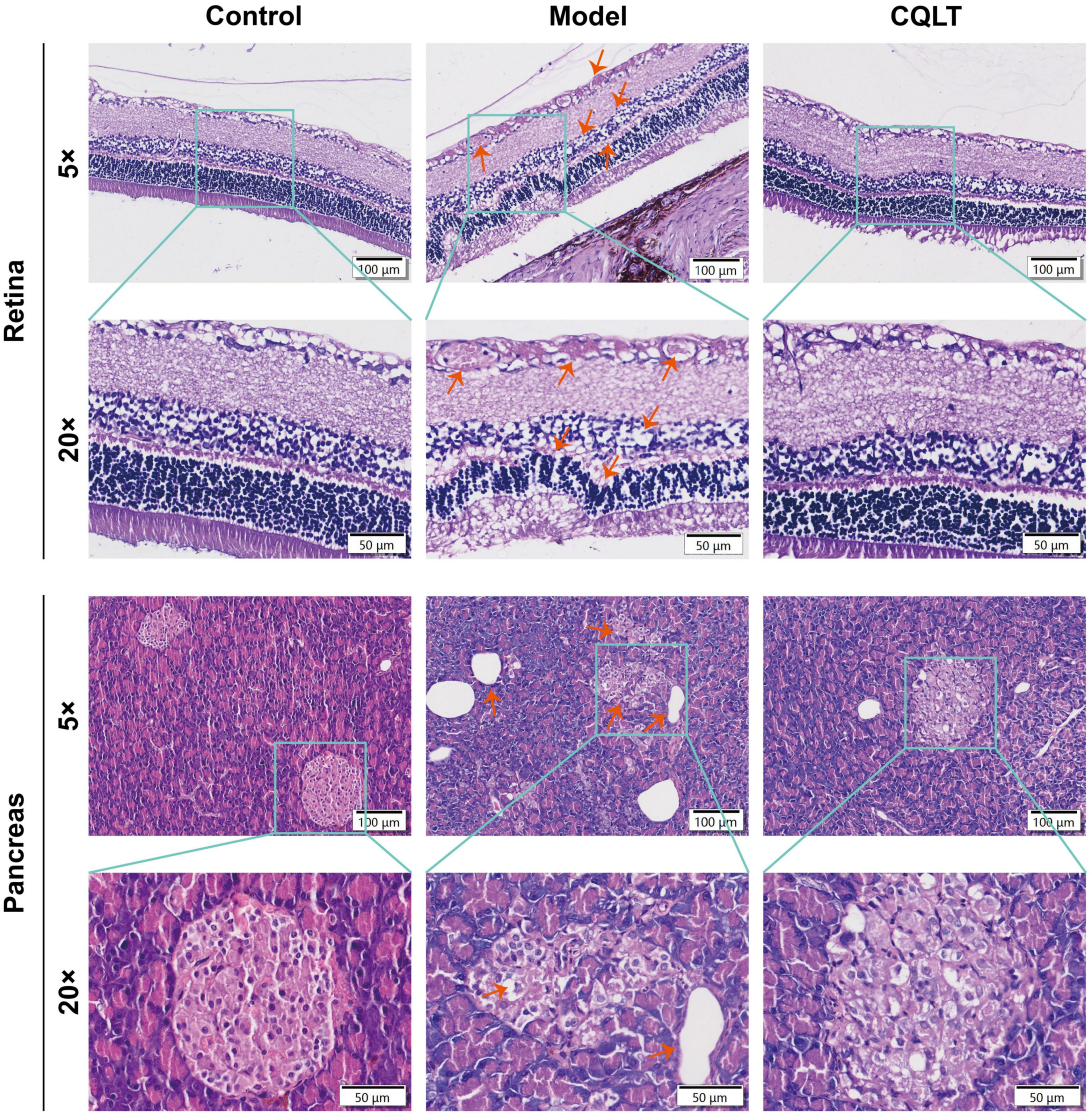
CQLT ameliorated retinal and pancreatic damage in DR rats.

In the sixth week of drug administration, the retina of the model group rats showed DR clinical features such as exudation, vascular proliferation, and cell vacuolar degeneration compared with the control group, indicating that the DR model was successfully established (Supplementary Figure S1). After the end of CQLT treatment, the results of pathological examination of the retina and pancreas of rats are shown in Figure 1. The retinas of control rats were structurally normal and aligned, and no pathological changes were observed. Compared with the control group, the retinas of DR rats showed more exudation, in which the exudation of the inner border layer was obvious; the microvessels were abnormally proliferated, the number of cells in the inner and outer granular layers was reduced, and the cells appeared to be vacuolated and degenerated; the number of ganglion cells was less, and their distributional spacing was increased; the inner and outer tufted layers were thinned, and the outer tufted layer was in the state of degeneration. Compared with the model group, the phenomena of abnormal retinal microvascular proliferation and exudation were suppressed in the CQLT group; the phenomenon of vacuolar degeneration of ganglion cells became fewer and densely distributed; and the number of cells in the inner and outer granular layer was significantly increased. These results indicated that CQLT improved retinal exudation, microvascular proliferation, and cell vacuolar degeneration in DR rats and normalized retinal structure and function. The islet tissue in the pancreas of the control group was regularly rounded, and the islet cells had a normal morphology. In the model group, the amount of islet tissue decreased, the cross-section of the islet became smaller, and the cells in the islets showed long shuttle-shaped changes. In the CQLT group, vacuolar degeneration of islet cells was inhibited, and islet morphology was improved.

### 3.2 CQLT suppressed retinal nerve damage indicator expression

**Figure 2:**
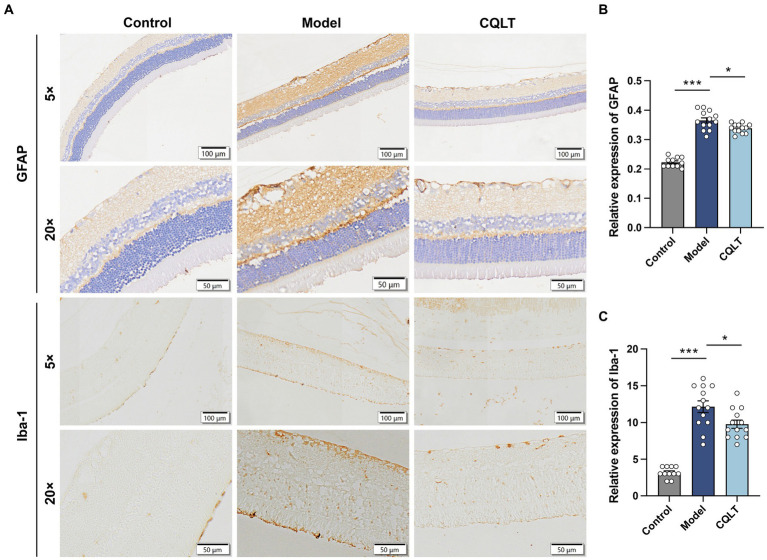
CQLT ameliorated retinal neurodegeneration in DR rats. The effects of CQLT on retinal GFAP and Iba-1 levels were observed under an optical microscope **(A)**. The immunohistochemical results were statistically analyzed using the NIS-ELEMNT BR image analysis system **(B,C)**. ^*^p < 0.05 and ^***^p < 0.001.

The intensity of GFAP positivity was weak in the control group; the intensity of expression was high in the model group, with dark brownish-yellow coloration in the inner border membrane layer and nerve fiber layer and brownish-yellow coloration in the inner and outer plexiform layers (Figure 2A). In the control group, the number of Iba-1 positive cells was small, and the cells were round. In the model group, the Iba-1 positive cells were oval or polygonal, with several foot processes visible in the cells, and the foot process sections of brown-yellow scattered positive cells were visible in the section. Iba-1 positive cells were mainly distributed in the ganglion cell layer, inner plexiform layer, and inner granular layer; the number of Iba-1 positive cells in the outer plexiform layer and outer granular layer was negligible (Figure 2A). Compared with the control group, the retinal GFAP and Iba-1 levels of DR rats were significantly increased; compared with the model group, the retinal GFAP and Iba-1 levels of rats in the CQLT group were significantly decreased (Figures 2B,C).

### 3.3 Effects of CQLT on metabolites in DR rats

**Figure 3:**
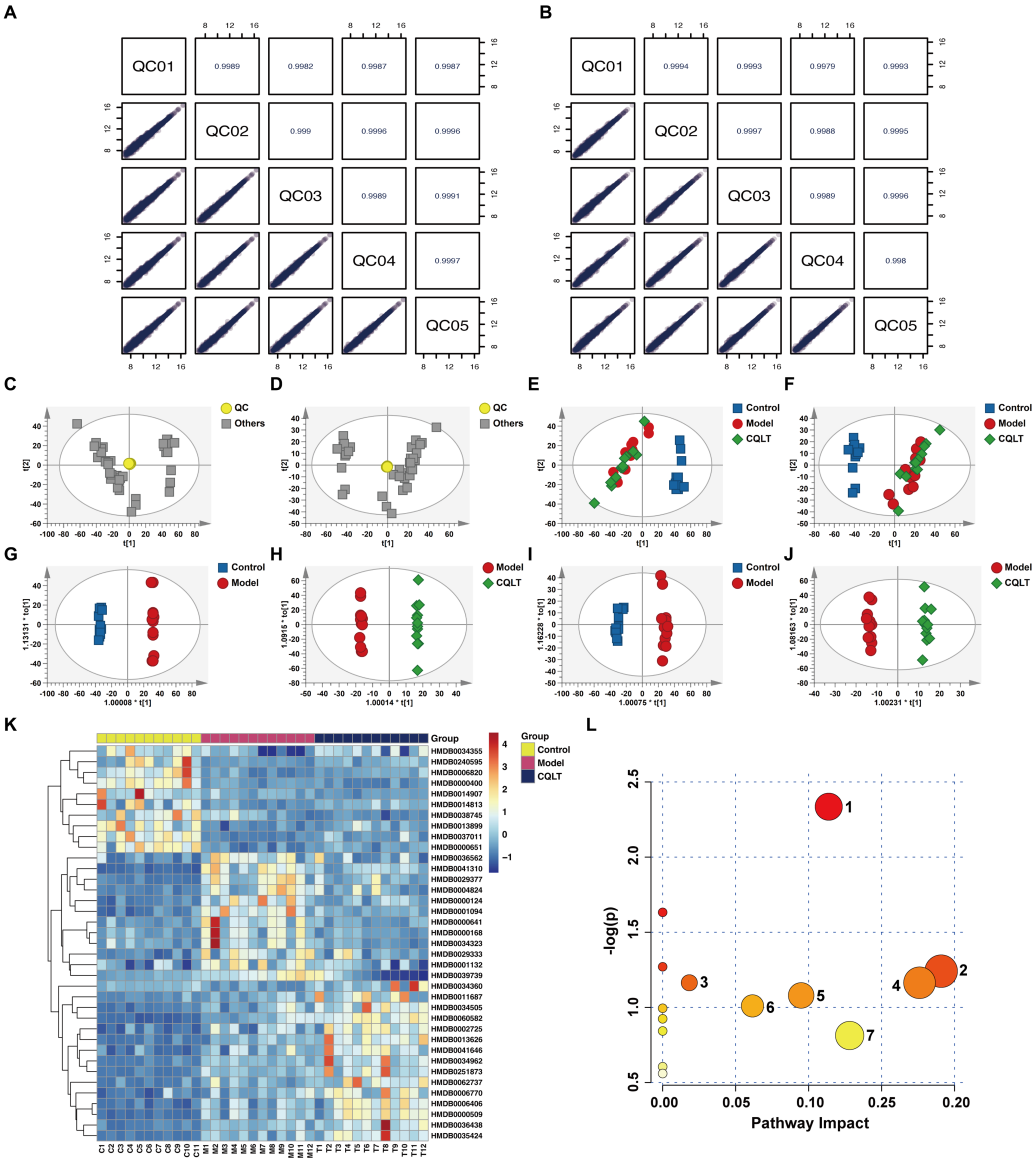
CQLT changed the fecal metabolite profile in DR rats. Correlation plots of QC samples in positive **(A)** and negative **(B)** ion modes. PCA in positive **(C,E)** and negative **(D,F)** ion modes. OPLS-DA in positive **(G,H)** and negative **(I,J)** ion modes. Heatmap of differential metabolites **(K)** and bubble map of metabolic pathways **(L)** for CQLT treatment of DR. Each number in the bubble map represents: **1:** alanine, aspartate and glutamate metabolism; **2:** nicotinate and nicotinamide metabolism; **3:** starch and sucrose metabolism; **4:** fructose and mannose metabolism; **5:** pentose phosphate pathway; **6:** glycolysis/gluconeogenesis; **7:** amino sugar and nucleotide sugar metabolism.

The total ion current (TIC) of different quality control (QC) samples was analyzed by mass spectrometry. The results showed high curve overlap for metabolite detection TIC, indicating good signal stability of mass spectrometry for the same sample at different times (Supplementary Figure S2). Pearson correlation analysis of the QC samples showed that |*r*| were all in the range of 0.997 to 1, indicating that the detection system is stable and the data quality is high (Figures 3A,B). PCA plot shows the approximate distance relationship between samples and groups (Figures 3C–F). The results showed a trend of group separation between the control group and the model and CQLT groups, respectively, but the metabolic profile separation between the model and CQLT groups was insignificant. Therefore, the OPLS-DA model was constructed for further analysis. The results showed that the metabolic profiles of these three data sets were separated (Figures 3G–J). The modeling data of OPLS-DA shows that the data of this model is effective, reliable, and has good predictive ability (Supplementary Table S1). The OPLS-DA model was validated, and the intercepts of the regression lines of *Q*^2^ on the vertical axis were all less than 0, indicating no overfitting in this model (Supplementary Figure S3).

Comparing the model group with the control group and the CQLT group with the model group, the differential metabolites are consistent with *p* < 0.05 and VIP >1, which can be used for further analysis. Fold Change (FC) >1.5 indicates up-regulation, FC <0.66 indicates down-regulation, and 37 differential metabolites were screened. Compared with the control group, 11 differential metabolites were up-regulated, and 26 differential metabolites were down-regulated in the model group; CQLT reversed the changing trend of 20 differential metabolites compared with the model group (Supplementary Table S2). These include metabolites such as l-glutamine, fructose-6-phosphate, and asparagine. Cluster analysis was performed on differential metabolites to show their changing trends (Figure 3K). The screened differential metabolites were imported into MetaboAnalyst 6.0 for metabolic pathway analysis. The metabolic pathways obtained include alanine, aspartate, and glutamate metabolism, nicotinate and nicotinamide metabolism, glycolysis/gluconeogenesis, starch and sucrose metabolism, fructose and mannose metabolism, amino sugar and nucleotide sugar metabolism, and pentose phosphate pathway (Figure 3L).

### 3.4 CQLT alleviated intestinal flora disorders in DR rats

#### 3.4.1 Alpha and beta diversity analysis

**Figure 4:**
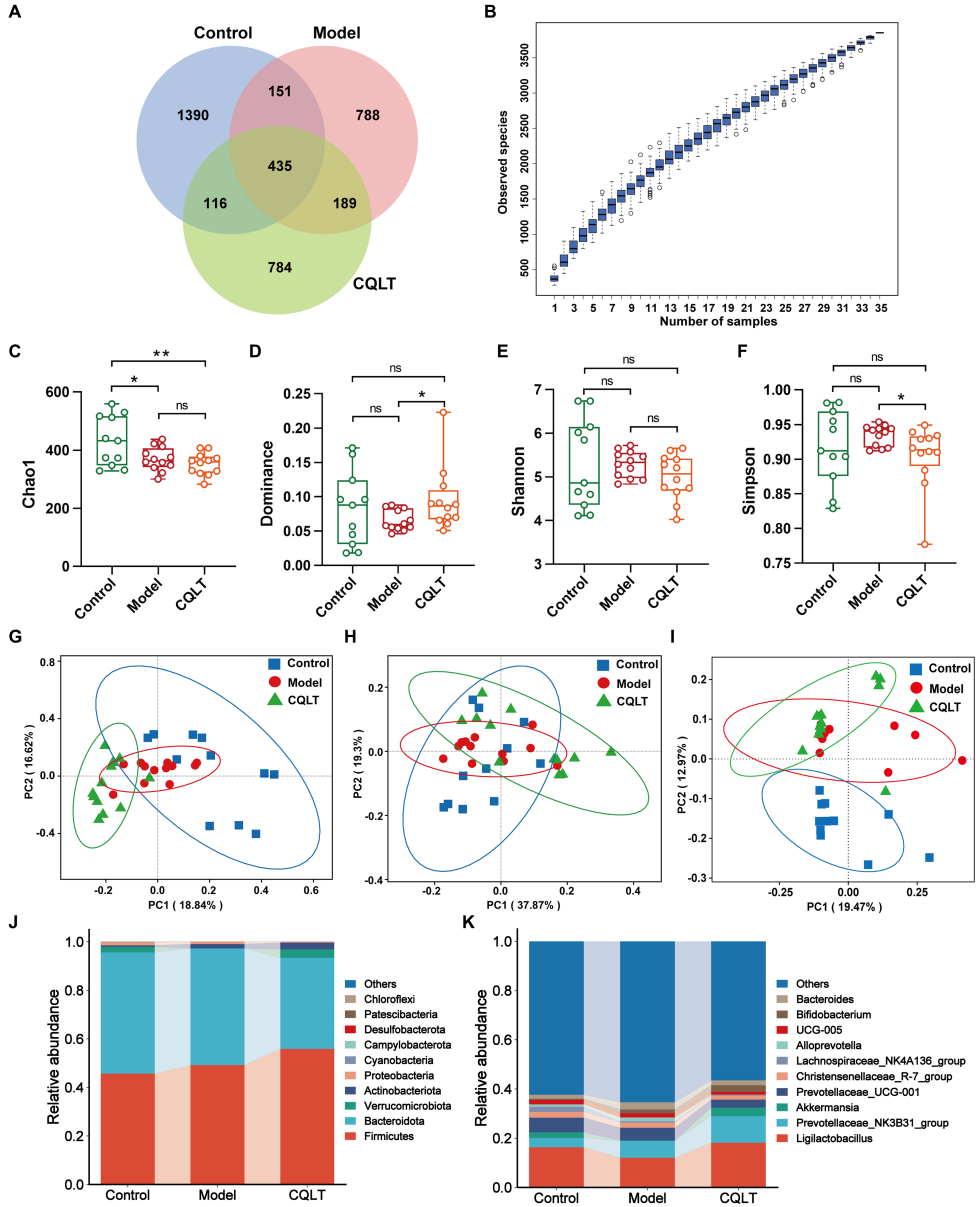
CQLT alleviated intestinal flora disorders in DR rats. Venn diagrams **(A)** analyze the ASVs data in each group. Species cumulative box plots of alpha diversity **(B)**. Chao1 **(C)**, Dominance **(D)**, Shannon **(E)**, and Simpson **(F)** indexes of each group. PCoA analysis based on Bray–Curtis **(G)**, weighted UniFrac **(H)**, and unweighted UniFrac **(I)** distances. Bar plots of relative abundance of intestinal flora at the phylum **(J)** and genus **(K)** levels. ^*^p < 0.05 and ^**^p < 0.01.

Analyze ASVs data in each group of samples. The numbers of ASVs in the control group, model group, and CQLT group were 1,390, 788, and 784, respectively; the three groups shared 435 ASVs (Figure 4A). In the alpha diversity index, the Chao1, Dominance, Shannon, and Simpson indices are used to characterize the richness and diversity of intestinal flora (Li et al., 2022). The species accumulation curve allows us to judge that the sample size of this experiment is sufficient and rich in species (Figure 4B). The Chao1 index was significantly lower in the model group compared to the control group (Figure 4C). Compared with the model group, the dominance index significantly increased, and the Simpson index significantly decreased in the CQLT group (Figures 4D–F). The results showed that CQLT could significantly change the richness and diversity of intestinal flora in DR rats. Characterization of beta diversity of intestinal flora by principal coordinate analysis (PCoA) based on Bray–Curtis, weighted UniFrac, and unweighted UniFrac distances (Guo et al., 2023). In the PCoA analysis of Bray–Curtis distances, PC1 and PC2 contributed 18.84 and 16.62%, respectively (Figure 4G). In the PCoA based on weighted UniFrac and unweighted UniFrac distances, the structure of the intestinal flora was well separated between groups and aggregated with each other within groups (Figures 4H,I). The results showed that the structure and composition of the intestinal flora of the three groups of rats were different, and they each had a unique microbial structure.

#### 3.4.2 Intestinal flora abundance analysis

Based on the ASVs annotation results, the relative abundance of species at the phylum and genus level is shown in Figures 4J,K. *Bacteroidota* and *Firmicutes* were the dominant intestinal flora in the control, model, and CQLT groups at the phylum level. Compared with the control group, the relative abundance of *Firmicutes* increased, and the relative abundance of *Bacteroidota* decreased in the model and CQLT groups. At the genus level, *Ligilactobacillus*, *Prevotellaceae_NK3B31_group*, *Christensenellaceae_R-7_group*, and *Prevotellaceae_UCG-001* were the dominant intestinal flora in the three experimental groups. *Akkermansia* is peculiar to the control group and CQLT group. Compared with the control group, the abundance of *Ligilactobacillus* and *Akkermansia* in the model group decreased, while the CQLT group could reverse this change and make their abundance tend to normal.

#### 3.4.3 Difference analysis of intestinal flora

**Figure 5:**
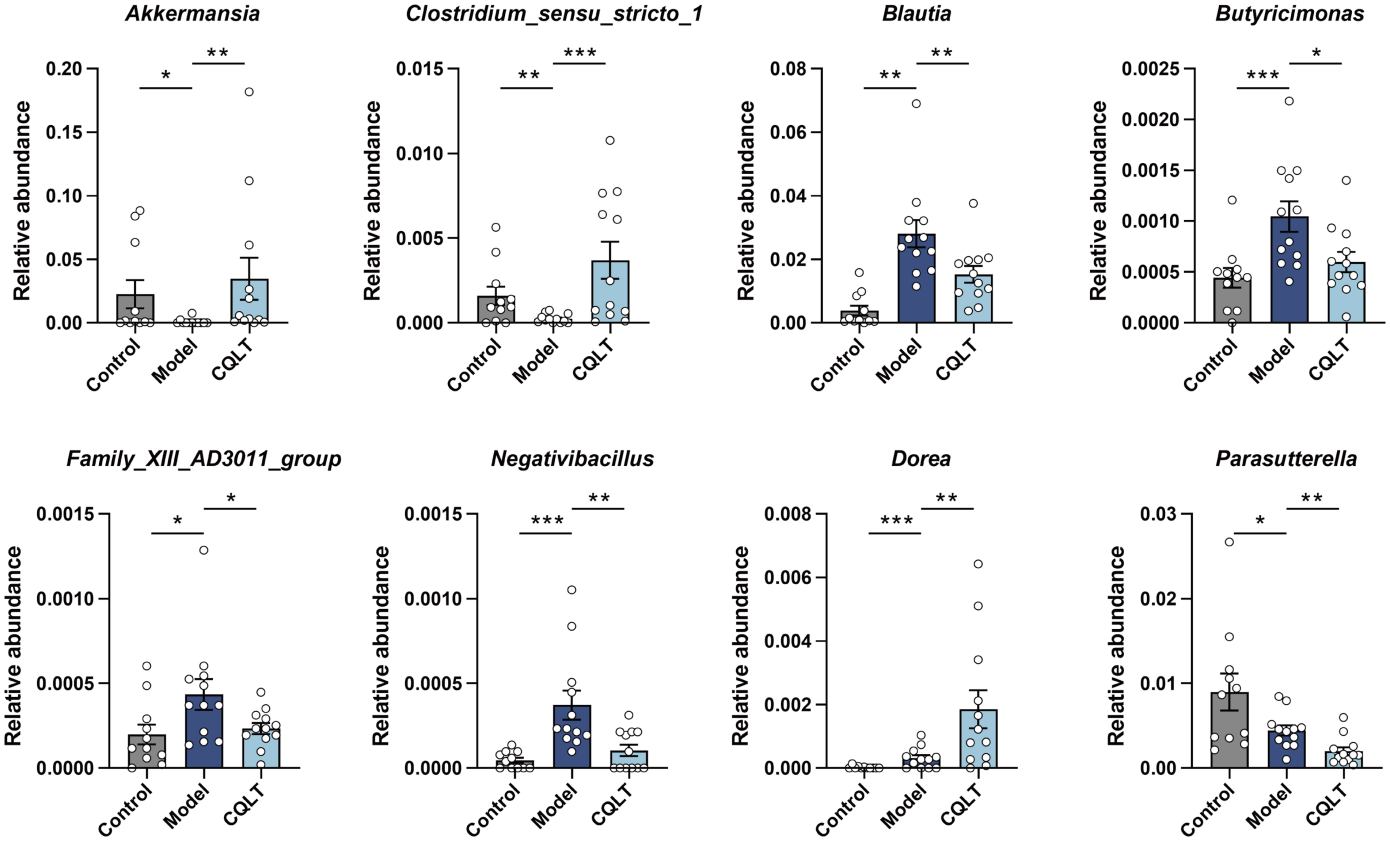
Analysis of differential intestinal flora at the genus level. ^*^p < 0.05, ^**^p < 0.01, and ^***^p < 0.001.

Whether there are significant differences in the intestinal flora of each group is often determined by ANOSIM and MRPP analysis. The results showed that the between-group differences were greater than the within-group differences, and the *p*-values were all less than 0.05, indicating statistical significance (Supplementary Table S3). This shows significant differences in intestinal flora between the control, model, and CQLT groups. The intestinal flora with significant differences between groups was analyzed using the MetaStat method. The results showed significant changes in the abundance of 8 intestinal flora at the genus level (Figure 5). *Akkermansia* and *Clostridium_sensu_stricto_1* abundance was significantly lower in the model group compared to the control group; the abundance of these intestinal microbiota genera was significantly higher and tended to normalize in the CQLT group compared to the model group. Compared with the control group, the abundance of *Blautia*, *Butyricimonas*, *Family_XIII_AD3011_group*, and *Negativibacillus* in the model group was significantly increased, while CQLT could significantly reduce the levels of these intestinal microbiota genera.

#### 3.4.4 Functional analysis of intestinal flora

**Figure 6:**
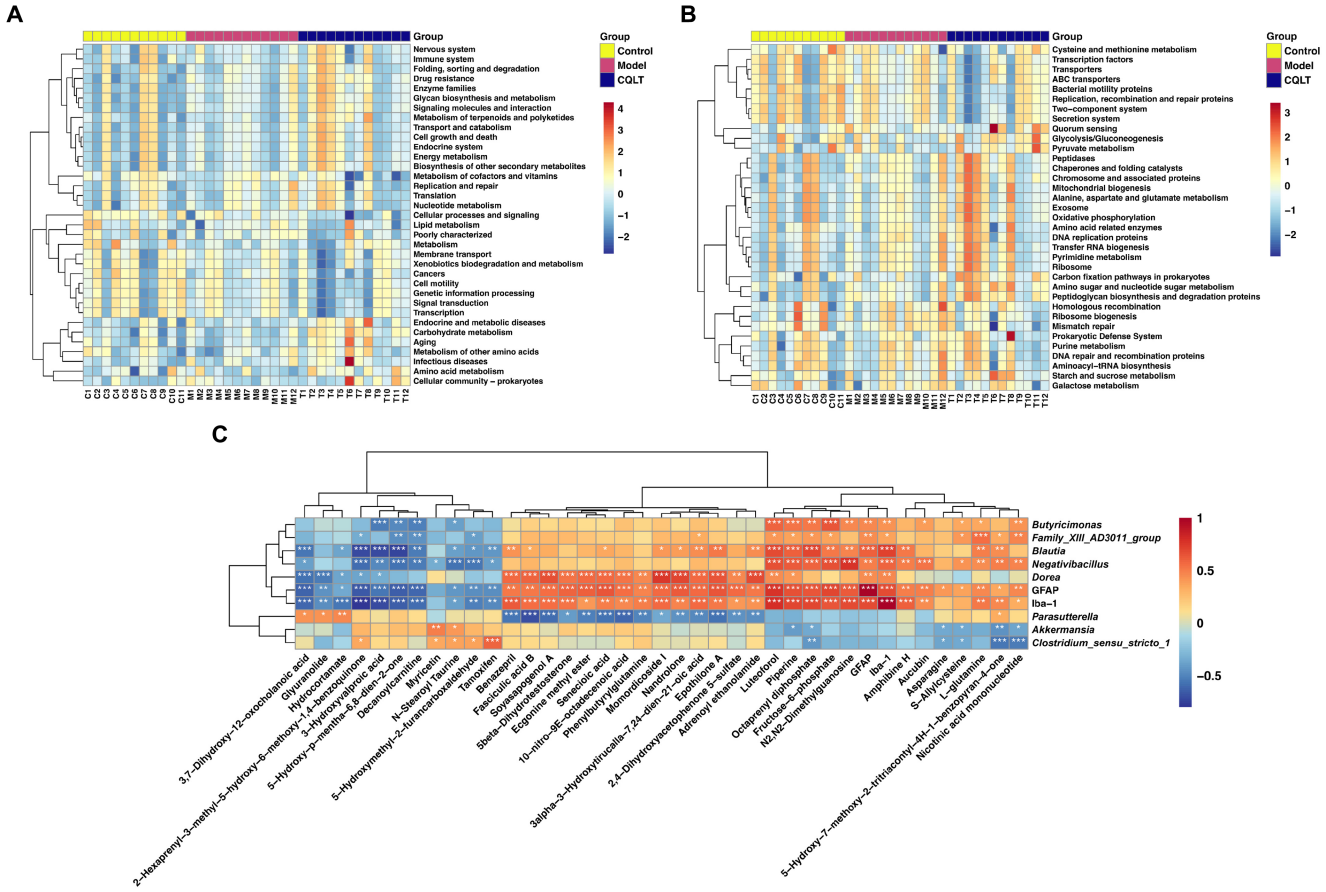
Functional annotation of intestinal flora and correlation analysis between retinal nerve damage indicators, intestinal flora, and metabolites. KEGG functional enrichment analysis of intestinal flora was performed in level 1 **(A)** and level 2 **(B)** databases, respectively. Spearman correlation analysis of retinal nerve damage indicators, significantly different metabolites, and intestinal microbiota genera **(C)**. ^*^p < 0.05, ^**^p < 0.01, and ^***^p < 0.001.

Intestinal flora function was analyzed using the Tax4Fun R package based on the 16S Silva database, and heat maps were created based on the functional annotations and abundance information of the samples in the database. Kyoto Encyclopedia of Genes and Genomes (KEGG) functional enrichment analysis was performed at the level 2 database, and the results showed that energy metabolism, lipid metabolism, amino acid metabolism, nucleotide metabolism, and carbohydrate metabolism were the main metabolic pathways involved in the intestinal flora in this study (Figure 6A). KEGG functional enrichment analysis was performed at the level 3 database, and the results showed that glycolysis/gluconeogenesis, alanine, aspartate and glutamate metabolism, starch and sucrose metabolism, amino sugar and nucleotide sugar metabolism, Purine metabolism, cysteine and methionine metabolism, and galactose metabolism were the main metabolic pathways involved in the intestinal flora in this study (Figure 6B).

### 3.5 Correlation analysis between metabolites, intestinal flora, and retinal nerve damage indices

Interactions among retinal nerve damage indices, 37 metabolites, and 8 intestinal flora genera were further focused by Spearman correlation analysis (Figure 6C). L-glutamine, fructose-6-phosphate, piperine, luteoforol, and octaprenyl diphosphate were significantly positively correlated with *Butyricimonas*, *Family_XIII_AD3011_group*, *Blautia* and *Negativibacillus*. Asparagine, s-allylcysteine, nicotinic acid mononucleotide, and octaprenyl diphosphate were significantly negatively correlated with *Akkermansia* and *Clostridium_sensu_stricto_1*. GFAP was significantly correlated with 36 metabolites, and Iba-1 was significantly correlated with 34 metabolites. GFAP and Iba-1 were significantly positively correlated with 5 intestinal flora genera. The specific correlation analysis results are shown in Supplementary Table S4.

### 3.6 Effects of CQLT on gluconeogenesis

**Figure 7:**
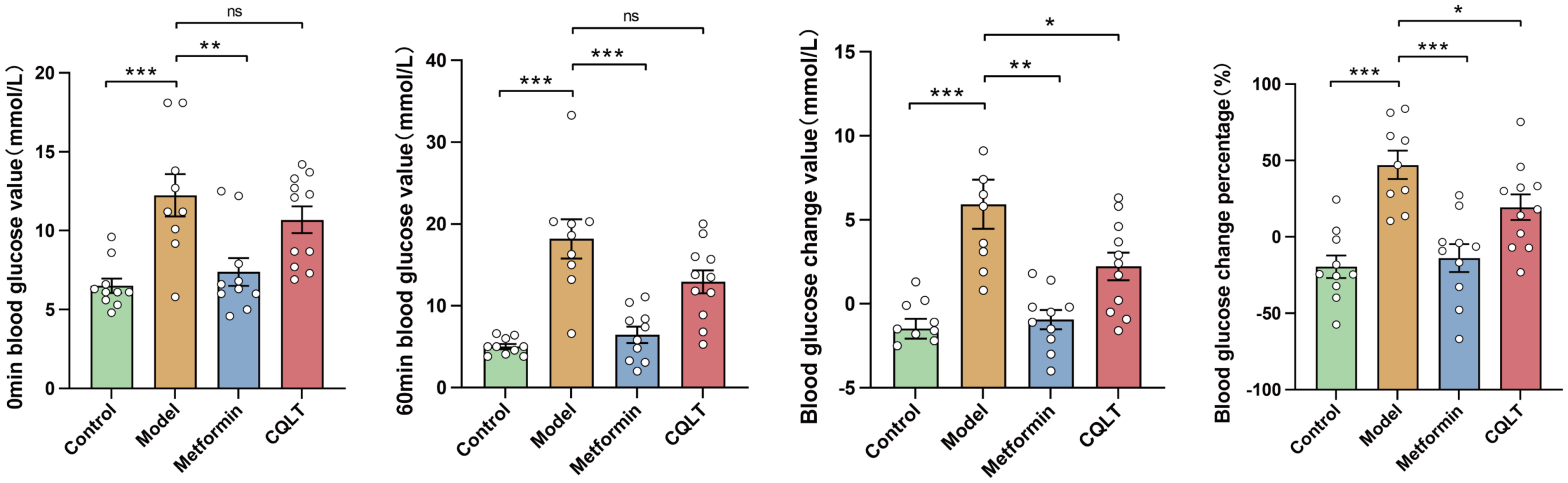
CQLT inhibited the gluconeogenesis process. ^*^p < 0.05, ^**^p < 0.01, and ^***^p < 0.001.

The mice were fasted for 12 h, and after being given the corresponding drug solution for 1 h (0 min blood glucose), compared with the model group, the blood glucose of the metformin group was significantly reduced. After 1 h of intraperitoneal injection of l-alanine (60 min blood glucose), compared with the model group, the blood glucose of the metformin group was significantly reduced; the blood glucose of the CQLT group tended to decrease, but it was not statistically significant. The value and percentage of change in blood glucose were significantly lower in the CQLT group compared to the model group (Figure 7).

## 4 Discussion

DR is one of the most common and severe complications of diabetes, which is characterized by increased retinal vascular permeability, retinal neurodegeneration, capillary obstruction, basement membrane thickening, and neovascularization (Wang and Lo, 2018; Liu et al., 2022a). If left untreated, it can lead to vitreous hemorrhage and neovascular glaucoma, resulting in significant impairment of vision (Shaikh et al., 2023; Tang et al., 2023). Vascular endothelial growth factor (VEGF), interleukin 6 (IL-6), tumor necrosis factor-alpha (TNF-α), reactive oxygen species (ROS), mechanistic target of rapamycin (mTOR), advanced glycation end products (AGEs), and protein kinase C (PKC) are currently considered key biological targets for the treatment of DR in preclinical studies (Safi et al., 2014; Yang et al., 2021; Lee et al., 2022). Most of these targets aim to intervene in the progression of DR through anti-inflammation, anti-oxidation, and anti-angiogenesis. Notably, many studies have focused on retinal microangiopathy, and retinal neuroprotection should be emphasized as a new approach to treating DR (Simó and Hernández, 2015; Liang et al., 2023). In this study, we aimed to explore a novel TCM for the treatment of DR, and we evaluated the mechanism of CQLT for the treatment of DR by pharmacodynamic studies, metabolomics, and intestinal microbiota. In addition, correlations between indicators of retinal neurodegeneration and metabolites and intestinal flora were investigated.

As diagnostic techniques for DR have improved, many researchers have found that severe retinal nerve damage exists early in the course of DR and that nerve damage is highly likely to precede and coexist with microangiopathy (Cohen and Gardner, 2016; Zafar et al., 2019). This also suggests that DR is not a simple microvascular disease but a more complex complication of diabetes and that retinal neurodegeneration is an early event in DR rather than the result of vascular injury (Kim et al., 2018; Mohammed et al., 2020). The disruption of retinal neurovascular unit (NVU) integrity is an important factor in the pathogenesis of DR. NVU is mainly composed of nerve cells, Müller cells, and microglia, which maintain the integrity of the blood-retinal barrier and thus maintain the function of the entire retina (Lechner et al., 2022; Wang and Cepko, 2022). GFAP is the signature protein of glial cells and the characteristic index of Müller cells from quiescence to injury (Bringmann et al., 2006; Zeng et al., 2022). Overexpression of GFAP in Müller cells is considered a marker of retinal damage (Li et al., 2002; Bahr et al., 2019). Iba-1 is a surface marker for microglia, which are first-responder cells to retinal nerve cell injury and are involved in damage repair and inflammatory states in the retina (McVicar et al., 2011; Zhang et al., 2019b; Xie et al., 2021). Our experimental data showed that retinal GFAP and Iba-1 levels were significantly increased in DR Rats. After intervention with CQLT, the expressions of retinal GFAP and Iba-1 were significantly reduced; this indicates that CQLT has a certain protective effect on retinal nerve damage in DR rats. Under normal physiological conditions, nerve growth factor (NGF), as a neurotrophic factor, binds to its tyrosine kinase receptor and activates protective signal transduction pathways. The decreased NGF synthesis in DR patients activates the apoptosis pathway, promotes retinal ganglion cells (RGCs) apoptosis, and leads to retinal nerve tissue degeneration (Wang Q. C. et al., 2020). Pigment epithelium-derived factor (PEDF) is a neurotrophic protective glycoprotein, and PEDF protects photoreceptors from damage by counteracting neurotoxicity (Xu et al., 2023). Retinal PEDF achieves DR neuroprotection by inhibiting glutamine synthetase activity in Müller cells (Shen et al., 2011). Moreover, downregulation of the neuroprotective factors somatostatin (SST) and glucagon-like peptide 1 (GLP-1) may predominate in the early stages of DR (Fragiotta et al., 2022). In short, targets such as NGF, RGCs, PEDF, SST, and GLP-1 deserve attention and may become potential therapeutic targets for treating DR nerve damage.

Metabolomics technology was used to study the mechanism of action of CQLT in treating DR at the metabolite level. We screened to obtain 37 differential metabolites shared by the control, model, and CQLT groups. These included significant differential metabolites such as l-glutamine, asparagine, and fructose-6-phosphate. Metabolic pathways of differential metabolites were analyzed, and the obtained metabolic pathways included alanine, aspartate and glutamate metabolism, nicotinate and nicotinamide metabolism, glycolysis/gluconeogenesis, starch and sucrose metabolism, fructose and mannose metabolism, amino sugar and nucleotide sugar metabolism, and pentose phosphate pathway. This suggests that CQLT may affect the progression of DR through the above metabolic pathways. There is a close relationship between amino acid metabolism and retinopathy. Many studies have focused on the protective effects of amino acid metabolism on microvascular endothelial cells, and individuals with higher levels of tyrosine and alanine have a lower risk of developing diabetic microvascular disease (Welsh et al., 2018; Hou et al., 2021). One study of patients with proliferative diabetic retinopathy (PDR) showed significant increases in vitreous amino acid levels, suggesting a beneficial effect of amino acids on DR (Vidhya et al., 2018). Alanine may participate in and regulate glucose metabolism by stimulating N-methyl-D-aspartate receptors and thereby inhibiting insulin secretion, which is a potential pathogenesis of DR (Marquard et al., 2015). We performed the pathological examination of pancreatic tissues and found that islet cells in the pancreas of DR rats were severely damaged. After CQLT treatment, the vacuolar degeneration of the islet cells was inhibited, and the pancreatic morphology improved. This suggests that CQLT may improve the state of islet cells by regulating the level of metabolites and then making insulin secretion tend to be normal. Glutamate is an essential excitatory neurotransmitter in the retina, and the concentration of glutamate is also one of the most important indexes affecting DR (Rhee et al., 2018). Glutamine abnormalities indicate disturbances in the glutamate cycle, which may affect Müller cell activation and proliferation (Du et al., 2022). Studies have shown that increased glutamate concentrations in the retina cause neuronal damage in the central nervous system and retina (Saxena et al., 2016; Narayanan et al., 2019). This is consistent with the results of our immunohistochemical experiments, suggesting that CQLT may exert a protective effect on the retinal nerves of DR rats by affecting amino acid metabolism. L-glutamine and asparagine are involved in alanine, aspartate, and glutamate metabolism. We found that l-glutamine and asparagine levels were significantly lower in DR rats compared with the control group by metabolomics results, while l-glutamine and asparagine levels were significantly higher in the CQLT group compared with the model group. This suggests that CQLT may affect the progression of DR by regulating alanine, aspartate, and glutamate metabolism. The disorder of glucose and lipid metabolism is characterized by the imbalance of glucose and lipid metabolism in the main metabolic organs or tissues, which is one of the leading causes of chronic diseases such as obesity and type 2 diabetes (Hu et al., 2023). Abnormal glucose metabolism can cause retinal vascular endothelial cell death and intraocular neovascular disorder, ultimately leading to pathological neovascularization (Milovanova et al., 2008). We found that fructose-6-phosphate levels were significantly reduced in DR rats, and CQLT reversed this change. Meanwhile, fructose-6-phosphate is a key metabolite in amino sugar and nucleotide sugar metabolism, starch and sucrose metabolism, glycolysis/gluconeogenesis, as well as fructose and mannose metabolism, suggesting that CQLT may improve the body’s metabolism by regulating the above metabolic pathways, which thereby affects the progression of DR. Previous studies have found that metabolites related to the pentose phosphate pathway are increased in the plasma of DR patients, and the pentose phosphate pathway slows oxidative stress caused by increased flux of the polyol pathway (Chen et al., 2016). The present study found that the pentose phosphate pathway was disorganized in DR rats, which CQLT ameliorated.

The “gut-retinal axis” theory has received widespread attention recently, and the relationship between intestinal microbiota, diabetes, and retina has gradually become a research focus (Zhang and Mo, 2023). Although the relationship between changes in intestinal flora and DR has not yet been determined, recent studies have suggested that intestinal flora imbalance plays a vital role in the development of DR (Huang et al., 2021; Liu et al., 2022b). In this study, the richness, function, and difference of intestinal flora were analyzed to explore the mechanism of CQLT in the treatment of DR. Alpha diversity results showed that the microbial information in this study was rich and the sample size was sufficient; the abundance and diversity of the intestinal flora changed significantly between the control, model, and CQLT groups, and CQLT had significant regulatory effects on the intestinal flora of DR rats. Analysis of beta diversity analysis showed that unique microbial structures were present in the control, model, and CQLT groups, respectively. By MetaStat analysis, we found that at the genus level *Akkermansia*, *Parasutterella* and *Clostridium_sensu_stricto_1*, *Blautia*, *Butyricimonas*, *Negativibacillus*, *Dorea* and *Family_ XIII_AD3011_group* were the gut microbes shared by the control, model and CQLT groups and they were significantly different between groups. As a beneficial bacterium, *Akkermansia* is abundant in the intestinal microbiota of healthy individuals and plays an important role in glucose metabolism, lipid metabolism, intestinal immunity, and maintaining the integrity of the intestinal mucosal barrier (Zhang et al., 2019a; Kalia et al., 2022; Rodrigues et al., 2022). The abundance of *Akkermansia* was significantly reduced in DR patients compared with healthy groups (Zhou et al., 2021). *Clostridium_sensu_stricto_1* has been reported to be strongly associated with insulin resistance (IR) in type 2 diabetic mice, and IR may be an essential risk factor for DR independent of hyperglycemia (Mbata et al., 2017). In addition, the inner retinal and photoreceptor cell layers are thinner in patients with metabolic syndrome, which may indicate that insulin resistance can lead to retinal neuropathy and that patients with high levels of IR have a higher likelihood of developing retinopathy compared with those with low levels of insulin resistance (Suzuki et al., 2000; Bao et al., 2020; Pillar et al., 2020). A study of fecal samples from patients with DR in coastal southeast China found an increased abundance of *Negativibacillus* in patients with PDR (Gu et al., 2023). Previous studies are in agreement with our experimental results; in the present study, the abundance of *Akkermansia* and *Clostridium_sensu_stricto_1* was significantly reduced, and the abundance of *Negativibacillus* was significantly increased in DR rats, whereas, after treatment with CQLT, the abundance of *Akkermansia* and *Clostridium_sensu_stricto_1* was significantly increased, and the abundance of *Negativibacillus* was significantly reduced.

The correlation between different intestinal flora and metabolites was analyzed. The results showed that l-glutamine and fructose-6-phosphate were significantly positively correlated with *Butyricimonas*, *Family_XIII_AD3011_group*, *Blautia*, and *Negativibacillus*. Asparagine, s-allylcysteine, nicotinic acid mononucleotide, and octaprenyl diphosphate were significantly negatively correlated with *Akkermansia* and *Clostridium_sensu_stricto_1*. This suggests that CQLT may directly or indirectly influence some fecal metabolites to benefit DR by increasing *Akkermansia* and *Clostridium_sensu_stricto_1* abundance and decreasing *Negativibacillus* abundance. At the same time, we found that the abundance of *Blautia*, *Butyricimonas*, and *Family_XIII_AD3011_group* was significantly increased in DR rats; however, CQLT could reduce the abundance of these intestinal flora. Although the relationship between *Blautia*, *Butyricimonas*, *Family_XIII_AD3011_group*, and DR has not been accurately reported, it also suggests that CQLT may have an anti-DR effect by changing the abundance of these intestinal flora to affect the state of metabolites. In addition, the retinal nerve injury indicators GFAP and Iba-1 were overall correlated with metabolites and intestinal flora, which further demonstrated that CQLT had a protective effect on the retinal nerves of DR rats by remodeling intestinal flora and affecting the status of metabolites.

Functional prediction of each group of intestinal flora revealed that energy metabolism, lipid metabolism, amino acid metabolism, nucleotide metabolism, and carbohydrate metabolism were the major metabolic pathways involved in this study of intestinal flora. KEGG functional enrichment analysis was performed at the level 3 database, where glycolysis/gluconeogenesis, alanine, aspartate and glutamate metabolism, starch and sucrose metabolism were metabolic pathways shared by metabolomics and 16S rDNA analysis results. We focused on the gluconeogenesis pathway. Gluconeogenesis helps maintain systemic glucose homeostasis, and diabetic patients often have decreased hepatic glycogen storage and glycogenolysis rate while gluconeogenesis output increases (Magnusson et al., 1992). The increase in gluconeogenesis flux will cause the patient’s blood glucose level to remain high even when fasting; in the long run, high blood glucose will accelerate the progression of diabetic complications, and patients will face more severe dangers than high blood glucose levels, DR being one of them (Shah and Wondisford, 2023). One study found gluconeogenesis to be the glucose metabolic pathway associated with DR by comparing aqueous humor and vitreous in patients with DR and normal controls (Wang H. et al., 2020). Our experimental data indicate that CQLT effectively reduces the gluconeogenesis process in diabetic mice; this is consistent with metabolomics and gut microbiota results, demonstrating that CQLT produces anti-DR effects by inhibiting gluconeogenesis. We performed the pathological examination of the rat retina and found that DR rats had retinal microvascular proliferation and impaired retinal function and morphology; compared with the model group, retinal edema, exudation, and abnormal microvascular proliferation were suppressed in the CQLT group. The results suggest that CQLT may produce anti-DR effects by inhibiting retinal neovascularization and protecting retinal function and morphology.

**Figure 8:**
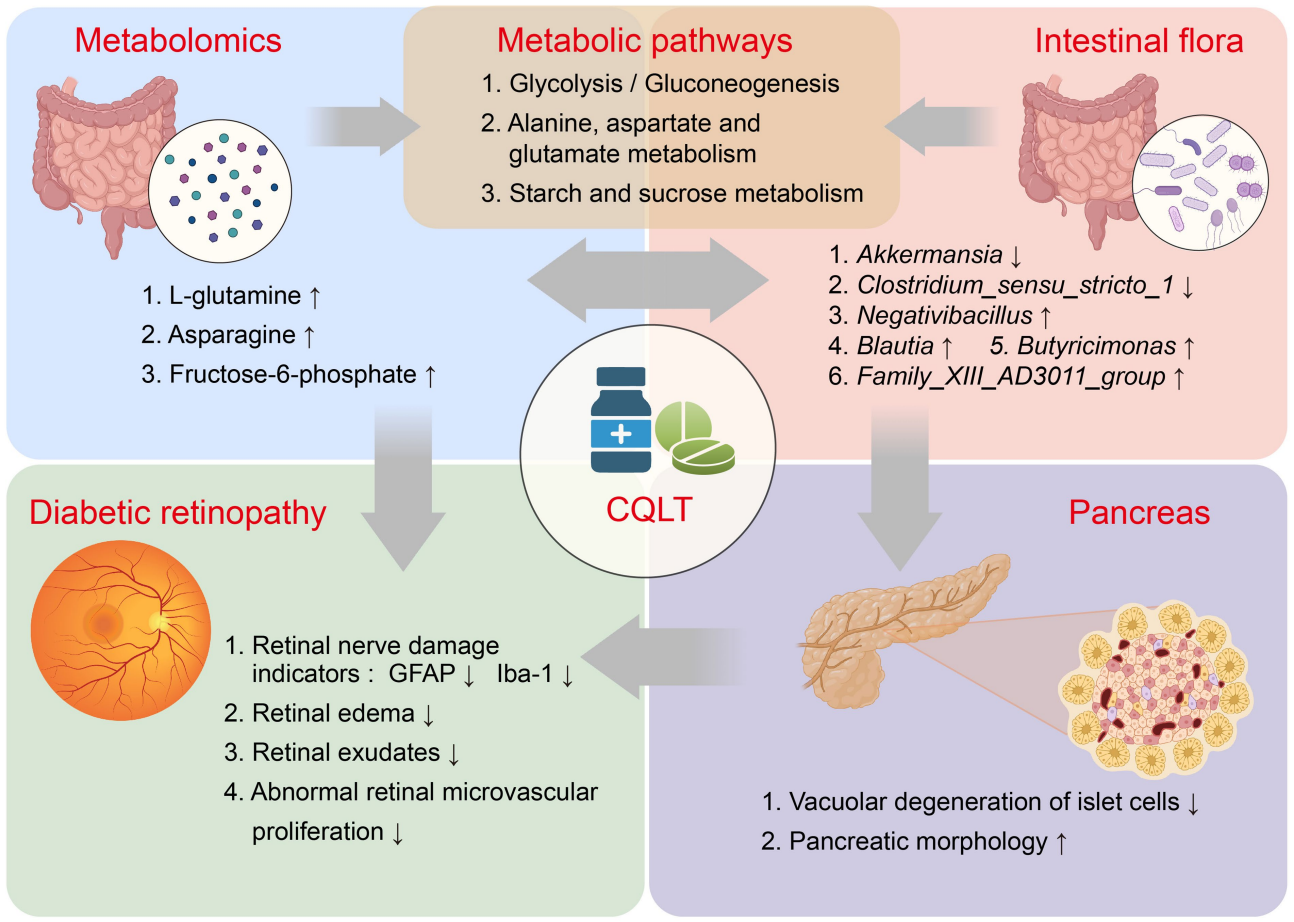
Potential mechanism of CQLT in treating DR.

Finally, we are also aware of the limitations of this study. Although metabolomics and gut microbiota allow for comprehensive screening and identification of substances for DR treatment with CQLT, the complexity of the pathogenesis of DR makes it difficult to delve into the specific relationship between metabolites and microbes. Further exploration of the interactions among metabolites, intestinal flora, and clinical pathogenic indicators of DR will help uncover the exact mechanism of action of CQLT in treating DR. In addition, the metabolic pathway of the CQLT treatment for DR remains to be validated accordingly. Nevertheless, our study also revealed the changes in metabolites and intestinal flora in ZDF rats with DR and explained the mechanism of action of CQLT in the treatment of DR. The mechanism of CQLT in treating DR is shown in Figure 8.

## 5 Conclusion

In this study, CQLT produced anti-DR effects by protecting the islet cell status, inhibiting retinal neovascularization, and attenuating exudation; meanwhile, CQLT also exerted protective effects on retinal nerves by inhibiting the expression of retinal Iba-1 and GFAP. L-glutamine, asparagine, and fructose-6-phosphate may be the potential characteristic metabolites of CQLT treatment for DR. CQLT alleviated intestinal flora disorders in DR rats, resulting in significantly decreased *Akkermansia* and *Clostridium_sensu_stricto_1* and significantly increased *Negativibacillus*, *Blautia*, *Butyricimonas*, and *Family_XIII_AD3011_group*. Furthermore, we found that CQLT inhibited the gluconeogenesis process in diabetic mice. In conclusion, CQLT regulates glycolysis/gluconeogenesis, alanine, aspartate and glutamate metabolism, starch and sucrose metabolism by changing the metabolic status of l-glutamine, fructose-6-phosphate, and asparagine and improving the intestinal flora structure of DR Rats. Eventually, CQLT produces anti-DR effects by protecting islet cell status, inhibiting retinal Iba-1 and GFAP expression, and protecting retinal function and morphology.

## Data Availability

The datasets presented in this study can be found in online repositories. The names of the repository/repositories and accession number(s) can be found in the article/Supplementary material.

## Footnotes

^1^ https://hmdb.ca/

^2^ https://www.metaboanalyst.ca/
