# Supplementary material for: Metabolomics combined with intestinal microbiota reveals the mechanism of compound Qilian tablets against diabetic retinopathy
Source: Front Microbiol. 2024 Aug 16;15:1453436. doi: 10.3389/fmicb.2024.1453436 (PMC11362098; doi:10.3389/fmicb.2024.1453436)
Supplement: Supplementary file 1 [file Data_Sheet_1.PDF]

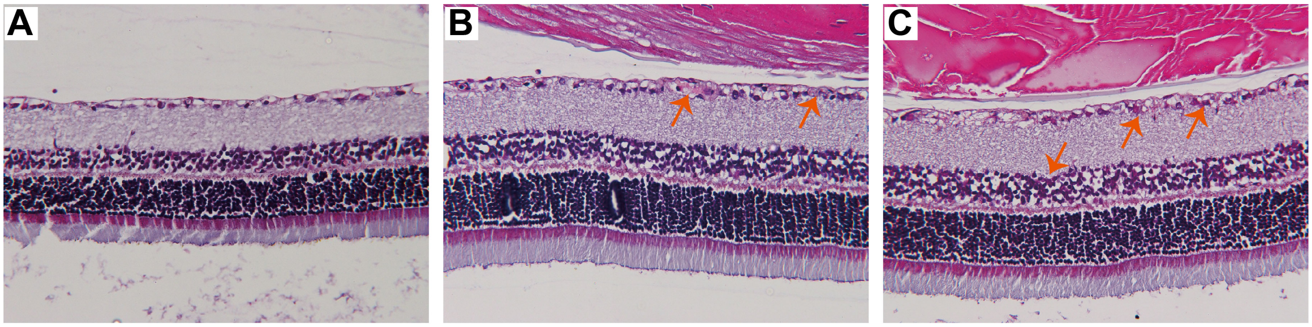

Supplementary Figure S1

Histopathologic examination of rat retina at the sixth week of CQLT treatment. H&E staining analysis of control (A) and model groups (B and C).

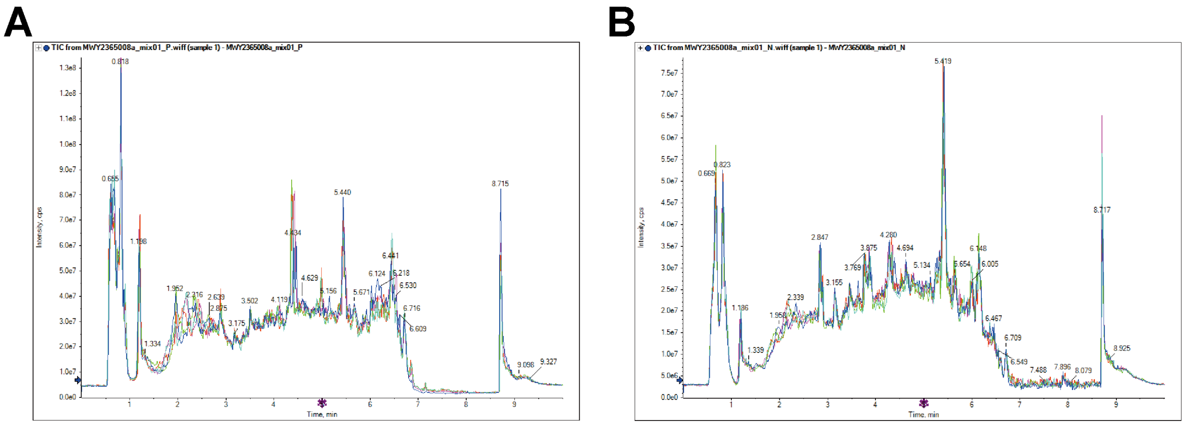

Supplementary Figure S2

Total ion current plots of different QC samples analyzed by mass spectrometry detection. Positive ion mode (A), negative ion mode (B).

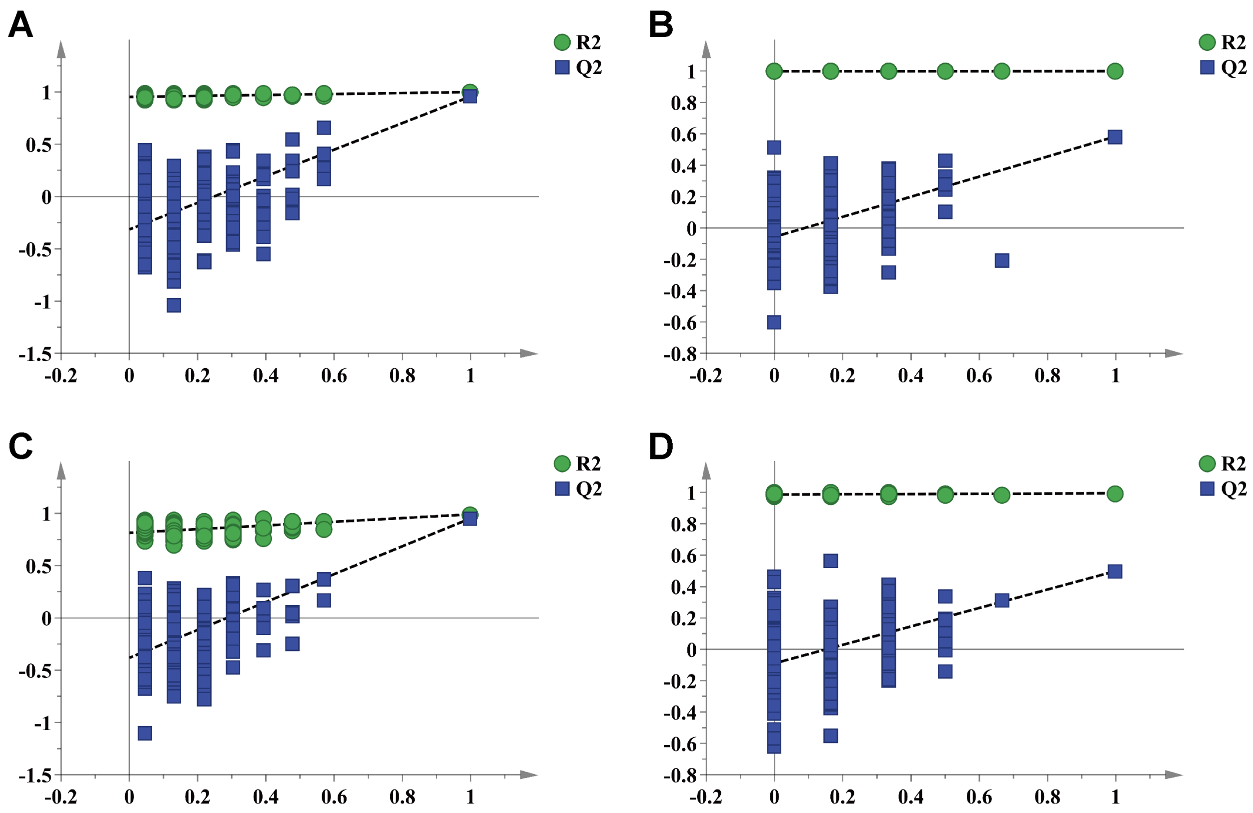

Supplementary Figure S3

OPLS-DA model validation. Model group *vs* control group (A) and CQLT group *vs* model group (B) in positive ion mode. Model group *vs* control group (C) and CQLT group *vs* model group (D) in negative ion mode.
