# Supplementary material for: Metabolomics combined with intestinal microbiota reveals the mechanism of compound Qilian tablets against diabetic retinopathy
Source: Front Microbiol. 2024 Aug 16;15:1453436. doi: 10.3389/fmicb.2024.1453436 (PMC11362098; doi:10.3389/fmicb.2024.1453436)
Supplement: Supplementary file 2 [file Data_Sheet_2.PDF]

Supplementary Table S1. Modeling data for OPLS-DA.

Acquisition mode	Model *vs* Control		CQLT *vs* Model	
	R_y^2	Q^2	R_y^2	Q^2
ESI+	0.999	0.957	0.999	0.583
ESI-	0.996	0.952	0.994	0.499

Supplementary Table S2. Fecal differential metabolites of CQLT treatment DR.

HMDB	Differential metabolite	Model *vs* Control		CQLT *vs* Model	
		FC	Trend	FC	Trend
HMDB0029377	Piperine	0.10	↓ ***	1.95	↑ #
HMDB0000124	Fructose-6-phosphate	0.25	↓ ***	1.93	↑ ##
HMDB0034355	5-Hydroxymethyl-2-furancarboxaldehyde	2.06	↑ **	0.62	↓ #

HMDB0006406	Ecgonine methyl ester	0.33	↓ ***	0.58	↓ ##
HMDB0062737	10-nitro-9E-octadecenoic acid	0.28	↓ **	0.47	↓ #
HMDB0006820	2-Hexaprenyl-3-methyl-5-hydroxy-6-methoxy-1,4-benzoquinone	5.78	↑ ***	0.55	↓ #
HMDB0000509	Senecioic acid	0.24	↓ ***	0.58	↓ ##
HMDB0000400	3,7-Dihydroxy-12-oxocholanoic acid	4.36	↑ ***	1.57	↑ #
HMDB0034962	3alpha-3-Hydroxytirucalla-7,24-dien-21-oic acid	0.18	↓ ***	0.52	↓ #
HMDB0013899	3-Hydroxyvalproic acid	3.18	↑ ***	0.64	↓ #
HMDB0006770	5beta-Dihydrotestosterone	0.53	↓ **	0.64	↓ #
HMDB0037011	5-Hydroxy-p-mentha-6,8-dien-2-one	4.61	↑ ***	0.55	↓ #
HMDB0001094	Octaprenyl diphosphate	0.23	↓ ***	2.21	↑ ###
HMDB0036562	Aucubin	0.31	↓ ***	1.94	↑ #
HMDB0000651	Decanoylcarnitine	4.38	↑ ***	0.65	↓ #

HMDB0036438	Fasciculic acid B	0.38	↓***	0.57	↓#
HMDB0038745	Glyuranolide	1.82	↑*	1.88	↑##
HMDB0014907	Hydrocortamate	2.34	↑*	1.52	↑#
HMDB0035424	Momordicoside I	0.14	↓***	0.52	↓#
HMDB0034360	Myricetin	2.04	↑**	0.25	↓#
HMDB0002725	Nandrolone	0.31	↓**	0.63	↓#
HMDB0240595	N-Stearoyl Taurine	5.04	↑**	0.51	↓##
HMDB0034505	Soyasapogenol A	0.42	↓**	0.57	↓##
HMDB0004824	N2, N2-Dimethylguanosine	0.18	↓***	1.80	↑#
HMDB0001132	Nicotinic acid mononucleotide	0.54	↓**	1.60	↑#
HMDB0029333	Amphibine H	0.25	↓***	1.75	↑#
HMDB0000168	Asparagine	0.29	↓**	3.71	↑##
HMDB0060582	Benazepril	0.24	↓***	0.55	↓###

HMDB0011687	Phenylbutyrylglutamine	0.32	↓ *	0.35	↓ #
HMDB0034323	S-Allylcysteine	0.39	↓ *	3.42	↑ ##
HMDB0013626	Adrenoyl ethanolamide	0.49	↓ ***	0.54	↓ ##
HMDB0041646	2,4-Dihydroxyacetophenone 5-sulfate	0.43	↓ **	0.62	↓ #
HMDB0039739	5-Hydroxy-7-methoxy-2-tritriacontyl-4H-1-benzopyran-4-one	0.63	↓ ***	1.88	↑ ###
HMDB0000641	L-glutamine	0.28	↓ **	3.21	↑ ##
HMDB0041310	Luteoforol	0.13	↓ ***	1.60	↑ ##
HMDB0014813	Tamoxifen	3.65	↓ **	0.54	↓ ###
HMDB0251873	Epothilone A	0.13	↓ ***	0.52	↓ #

↑ is up-regulated, ↓ is down-regulated; compared with the control group, $^{*}P < 0.05$, $^{**}P < 0.01$, $^{***}P < 0.001$; compared with the model group, $^{\#}P < 0.05$，$^{\#\#}P < 0.01$，$^{\#\#\#}P < 0.001$.

Supplementary Table S3(A). Analysis of differences between groups based on Anosim.

Group	*R*-value	*P*-value
C-M	0.40784	＜0.001
C-G	0.51453	＜0.001
M-G	0.39904	＜0.001

The *R*-value is between (-1, 1), and the *R*-value is greater than 0, indicating that the difference between groups is greater than within the group. The *R*-value is less than 0, indicating that the difference within the group is greater than between the groups. $P < 0.05$ indicates statistical significance.

Supplementary Table S3(B). Analysis of differences between groups based on MRPP.

Group	*A*-value	observed-delta	expected-delta	Significance
C-M	0.06934	0.57204	0.61466	0.001
C-G	0.08838	0.60627	0.66505	0.001
M-G	0.05960	0.52746	0.56088	0.001

The smaller the Observe Delta value, the smaller the difference within the group; the larger the Expected Delta value, the larger the difference between the groups. The *A*-value is greater than 0, indicating that the difference between groups is greater than within the group.

The *A*-value is less than 0, indicating that the difference within the group is greater than between the groups. $P < 0.05$ indicates statistical significance.

Supplementary Table S4. Correlation analysis between retinal nerve damage indicators, intestinal flora, and metabolites.

Data1	Data2	rho	*P*-value	relation
GFAP	GFAP	1	0	positive
Iba-1	Iba-1	1	0	positive
Iba-1	2-Hexaprenyl-3-methyl-5-hydroxy-6-methoxy-1,4-benzoquinone	-0.816783906	2.18119E-09	negtive
Iba-1	Luteoforol	0.796378381	1.05812E-08	positive
GFAP	Luteoforol	0.779943151	3.3357E-08	positive
Negativibacillus	N2, N2-Dimethylguanosine	0.774925557	4.64593E-08	positive
Dorea	Momordicoside I	0.747530117	2.47118E-07	positive
Blautia	5-Hydroxy-p-mentha-6,8-dien-2-one	-0.735014006	1.56309E-06	negtive
Blautia	2-Hexaprenyl-3-methyl-5-hydroxy-6-methoxy-1,4-benzoquinone	-0.721288515	2.53221E-06	negtive

Blautia	Iba-1	0.720666848	1.04914E-06	positive
Blautia	3-Hydroxyvalproic acid	-0.714565826	3.25124E-06	negtive
Iba-1	3-Hydroxyvalproic acid	-0.71363046	1.49135E-06	negtive
Parasutterella	Fasciculic acid B	-0.707892711	1.97177E-06	negtive
GFAP	Fructose-6-phosphate	0.706331802	2.12498E-06	positive
Iba-1	Piperine	0.703216606	2.46376E-06	positive
Iba-1	Octaprenyl diphosphate	0.701950056	2.61509E-06	positive
Dorea	Nandrolone	0.700935319	2.74241E-06	positive
Blautia	Octaprenyl diphosphate	0.700560224	5.55277E-06	positive
GFAP	Iba-1	0.688969948	4.73542E-06	positive
Iba-1	GFAP	0.688969948	4.73542E-06	positive
Blautia	Luteoforol	0.686554622	9.52028E-06	positive
Dorea	Adrenoyl ethanolamide	0.685116098	5.61578E-06	positive
Iba-1	N2, N2-Dimethylguanosine	0.67535251	8.5536E-06	positive
Negativibacillus	Fructose-6-phosphate	0.669616623	1.08736E-05	positive

Dorea	Soyasapogenol A	0.663832055	1.37794E-05	positive
GFAP	2-Hexaprenyl-3-methyl-5-hydroxy-6-methoxy-1,4-benzoquinone	-0.661518786	1.51269E-05	negtive
GFAP	Piperine	0.657304396	1.7893E-05	positive
Negativibacillus	Luteoforol	0.656791448	1.82593E-05	positive
GFAP	5-Hydroxy-p-mentha-6,8-dien-2-one	-0.654354323	2.00942E-05	negtive
Iba-1	5-Hydroxy-p-mentha-6,8-dien-2-one	-0.652836068	2.13202E-05	negtive
Butyricimonas	Fructose-6-phosphate	0.652375005	2.17057E-05	positive
GFAP	Epothilone A	0.651825689	2.21733E-05	positive
Dorea	Epothilone A	0.648588078	2.51194E-05	positive
Iba-1	Fructose-6-phosphate	0.647206958	2.64808E-05	positive
GFAP	Octaprenyl diphosphate	0.642975469	3.1078E-05	positive
GFAP	Senecioic acid	0.637918201	3.75127E-05	positive
Iba-1	Benazepril	0.630882538	4.84704E-05	positive
GFAP	3,7-Dihydroxy-12-oxocholanoic acid	-0.623589274	6.2806E-05	negtive

GFAP	N2, N2-Dimethylguanosine	0.622184477	6.59707E-05	positive
GFAP	Nandrolone	0.619093924	7.34445E-05	positive
Negativibacillus	Piperine	0.618600928	7.47049E-05	positive
GFAP	Ecgonine methyl ester	0.61656529	8.01177E-05	positive
Parasutterella	10-nitro-9E-octadecenoic acid	-0.613488341	8.89715E-05	negtive
Iba-1	3,7-Dihydroxy-12-oxocholanoic acid	-0.613150841	8.99943E-05	negtive
Blautia	Piperine	0.611764706	0.000130161	positive
Iba-1	Amphibine H	0.607943914	0.000107174	positive
Blautia	GFAP	0.604203079	0.000121279	positive
GFAP	Decanoylcarnitine	-0.602657803	0.000127577	negtive
Iba-1	Decanoylcarnitine	-0.598796609	0.000144615	negtive
Negativibacillus	Octaprenyl diphosphate	0.5945181	0.000165856	positive
GFAP	5beta-Dihydrotestosterone	0.593667103	0.0001704	positive
Parasutterella	Soyasapogenol A	-0.590657611	0.000187377	negtive

Negativibacillus	2-Hexaprenyl-3-methyl-5-hydroxy-6-methoxy-1,4-benzoquinone	-0.58938803	0.000194982	negtive
Butyricimonas	Luteoforol	0.588482608	0.000200574	positive
Clostridium_sensu_stricto_1	Tamoxifen	0.588368417	0.000201289	positive
Negativibacillus	Iba-1	0.585127169	0.000222568	positive
Dorea	Fasciculic acid B	0.581859726	0.000246034	positive
GFAP	2,4-Dihydroxyacetophenone 5-sulfate	0.581164412	0.000251304	positive
Dorea	3,7-Dihydroxy-12-oxocholanoic acid	-0.580709237	0.000254808	negtive
GFAP	Momordicoside I	0.579900095	0.000261145	positive
Negativibacillus	N-Stearoyl Taurine	-0.579697898	0.000262751	negtive
GFAP	3alpha-3-Hydroxytirucalla-7,24-dien-21-oic acid	0.577371461	0.000281867	positive
Dorea	Ecgonine methyl ester	0.573806304	0.000313577	positive
Parasutterella	Epothilone A	-0.573009316	0.000321086	negtive
Parasutterella	Senecioic acid	-0.571888789	0.000331915	negtive
Negativibacillus	5-Hydroxy-p-mentha-6,8-dien-2-one	-0.571860291	0.000332195	negtive

Dorea	Benazepril	0.570930082	0.000341438	positive
Iba-1	Momordicoside I	0.569665964	0.000354366	positive
Dorea	3alpha-3-Hydroxytirucalla-7,24-dien-21-oic acid	0.568341482	0.000368377	positive
Iba-1	3alpha-3-Hydroxytirucalla-7,24-dien-21-oic acid	0.567555047	0.000376928	positive
Dorea	Senecioic acid	0.565752882	0.000397194	positive
Clostridium_sensu_stricto_1	5-Hydroxy-7-methoxy-2-tritriacontyl-4H-1-benzopyran-4-one	-0.559742813	0.000471973	negtive
Blautia	3,7-Dihydroxy-12-oxocholanoic acid	-0.557703081	0.000615673	negtive
Iba-1	Soyasapogenol A	0.556156099	0.000522337	positive
Family_XIII_AD3011_group	L-glutamine	0.55524432	0.000535878	positive
Iba-1	Fasciculic acid B	0.552778633	0.000574069	positive
GFAP	Soyasapogenol A	0.549275526	0.000632479	positive
Parasutterella	Benazepril	-0.548217664	0.000651124	negtive
GFAP	3-Hydroxyvalproic acid	-0.54773025	0.000659877	negtive
Negativibacillus	5-Hydroxymethyl-2-furancarboxaldehyde	-0.547207455	0.000669382	negtive

GFAP	10-nitro-9E-octadecenoic acid	0.546465933	0.000683071	positive
Iba-1	Hydrocortamate	-0.546164428	0.000688707	negtive
Butyricimonas	3-Hydroxyvalproic acid	-0.542665033	0.000757206	negtive
Dorea	5beta-Dihydrotestosterone	0.541592617	0.000779371	positive
Dorea	Glyuranolide	-0.539004017	0.000835255	negtive
Blautia	Decanoylcarnitine	-0.53837535	0.001012269	negtive
Negativibacillus	Aucubin	0.538229833	0.000852642	positive
Clostridium_sensu_stricto_1	Nicotinic acid mononucleotide	-0.537431681	0.000870902	negtive
Butyricimonas	Piperine	0.536920322	0.000882781	positive
Iba-1	5beta-Dihydrotestosterone	0.536735668	0.000887106	positive
Dorea	10-nitro-9E-octadecenoic acid	0.532676328	0.000987029	positive
Negativibacillus	Decanoylcarnitine	-0.530249724	0.001051392	negtive
Butyricimonas	Decanoylcarnitine	-0.526271589	0.001164934	negtive
Butyricimonas	Octaprenyl diphosphate	0.524169866	0.001229173	positive

Iba-1	L-glutamine	0.521959254	0.001300084	positive
GFAP	Adrenoyl ethanolamide	0.519915274	0.001368826	positive
Iba-1	Epothilone A	0.519426154	0.001385742	positive
Blautia	Epothilone A	0.517366947	0.001685716	positive
Blautia	Amphibine H	0.516246499	0.001730736	positive
GFAP	Benazepril	0.514296087	0.001574541	positive
Parasutterella	Ecgonine methyl ester	-0.513201206	0.001617639	negtive
Blautia	Fructose-6-phosphate	0.511204482	0.001946686	positive
GFAP	Fasciculic acid B	0.511065055	0.001704713	positive
Iba-1	5-Hydroxymethyl-2-furancarboxaldehyde	-0.510138122	0.001743757	negtive
Negativibacillus	GFAP	0.506983322	0.001882578	positive
Negativibacillus	5-Hydroxy-7-methoxy-2-tritriacontyl-4H-1-benzopyran-4-one	0.505169382	0.001966699	positive
GFAP	Amphibine H	0.50474347	0.001986921	positive
Akkermansia	Myricetin	0.504693796	0.001989291	positive

Iba-1	Senecioic acid	0.504509012	0.001998131	positive
GFAP	Aucubin	0.500950518	0.00217518	positive
Negativibacillus	Amphibine H	0.498329289	0.002314219	positive
Parasutterella	Phenylbutyrylglutamine	-0.495973109	0.002445756	negtive
Blautia	L-glutamine	0.492156863	0.002991289	positive
Iba-1	Phenylbutyrylglutamine	0.491843513	0.002692073	positive
Parasutterella	3alpha-3-Hydroxytirucalla-7,24-dien-21-oic acid	-0.490650607	0.002767131	negtive
Negativibacillus	3-Hydroxyvalproic acid	-0.489066663	0.002869606	negtive
Iba-1	Nandrolone	0.488747503	0.002890649	positive
Iba-1	Tamoxifen	-0.487621681	0.002965954	negtive
Blautia	N2, N2-Dimethylguanosine	0.481792717	0.003743871	positive
Family_XIII_AD3011_ group	Nicotinic acid mononucleotide	0.481071531	0.003438834	positive
Butyricimonas	5-Hydroxy-p-mentha-6,8-dien-2-one	-0.479333095	0.003574819	negtive
Negativibacillus	Nicotinic acid mononucleotide	0.479234029	0.003582707	positive

GFAP	Phenylbutyrylglutamine	0.47439986	0.003986401	positive
Dorea	2,4-Dihydroxyacetophenone 5-sulfate	0.470837554	0.004308537	positive
GFAP	L-glutamine	0.468921153	0.004490963	positive
GFAP	Nicotinic acid mononucleotide	0.467375876	0.004642882	positive
Parasutterella	Adrenoyl ethanolamide	-0.466559284	0.004724939	negtive
GFAP	Tamoxifen	-0.466252039	0.004756135	negtive
Iba-1	5-Hydroxy-7-methoxy-2-tritriacontyl-4H-1-benzopyran-4-one	0.462431412	0.005159162	positive
Dorea	Phenylbutyrylglutamine	0.462208887	0.005183515	positive
Parasutterella	Hydrocortamate	0.461096717	0.005306723	positive
Iba-1	Glyuranolide	-0.459476129	0.005490764	negtive
GFAP	5-Hydroxymethyl-2-furancarboxaldehyde	-0.458806616	0.005568385	negtive
Dorea	Iba-1	0.458501194	0.005604106	positive
Iba-1	10-nitro-9E-octadecenoic acid	0.458491035	0.005605298	positive
Butyricimonas	N2, N2-Dimethylguanosine	0.455373446	0.005981412	positive

Family_XIII_AD3011_group	5-Hydroxy-p-mentha-6,8-dien-2-one	-0.455132087	0.006011413	negtive
Butyricimonas	GFAP	0.45499416	0.006028615	positive
Family_XIII_AD3011_group	Decanoylcarnitine	-0.451907183	0.006424843	negtive
GFAP	Glyuranolide	-0.451501673	0.006478515	negtive
Negativibacillus	L-glutamine	0.447883602	0.006974653	positive
Parasutterella	2,4-Dihydroxyacetophenone 5-sulfate	-0.446249738	0.007209149	negtive
Iba-1	Aucubin	0.44371462	0.007586338	positive
Dorea	Luteoforol	0.443225822	0.007660971	positive
Family_XIII_AD3011_group	GFAP	0.442726345	0.007737881	positive
Blautia	3alpha-3-Hydroxytirucalla-7,24-dien-21-oic acid	0.438655462	0.008926459	positive
Dorea	GFAP	0.438363941	0.008438106	positive
Akkermansia	5-Hydroxy-7-methoxy-2-tritriacontyl-4H-1-benzopyran-4-one	-0.438049417	0.008490614	negtive
Blautia	Adrenoyl ethanolamide	0.437254902	0.009166614	positive

Blautia	5-Hydroxy-7-methoxy-2-tritriacontyl-4H-1-benzopyran-4-one	0.436694678	0.009264216	positive
Parasutterella	Nandrolone	-0.436585196	0.008738741	negtive
Blautia	Benazepril	0.435854342	0.009412289	positive
Blautia	Tamoxifen	-0.433613445	0.00981711	negtive
Butyricimonas	Iba-1	0.433553334	0.00927221	positive
Butyricimonas	Nicotinic acid mononucleotide	0.433375406	0.009304359	positive
Iba-1	Adrenoyl ethanolamide	0.432597127	0.0094461	positive
Clostridium_sensu_stricto_1	Octaprenyl diphosphate	-0.430787274	0.009782828	negtive
Parasutterella	Glyuranolide	0.426500456	0.010621305	positive
Blautia	5-Hydroxymethyl-2-furancarboxaldehyde	-0.426330532	0.011237393	negtive
GFAP	N-Stearoyl Taurine	-0.42242238	0.011474726	negtive
Parasutterella	3,7-Dihydroxy-12-oxocholanoic acid	0.422298481	0.011501536	positive
Negativibacillus	S-Allylcysteine	0.419383214	0.012147843	positive
GFAP	Asparagine	0.416662714	0.012778383	positive

Akkermansia	N-Stearoyl Taurine	0.415505864	0.013054758	positive
Iba-1	Ecgonine methyl ester	0.415287613	0.013107459	positive
Dorea	Piperine	0.413600734	0.013520878	positive
Family_XIII_AD3011_group	Octaprenyl diphosphate	0.411665991	0.014008512	positive
Akkermansia	Piperine	-0.410715359	0.014253479	negtive
Iba-1	2,4-Dihydroxyacetophenone 5-sulfate	0.410643597	0.014272117	positive
Blautia	Momordicoside I	0.405602241	0.016275463	positive
Family_XIII_AD3011_group	S-Allylcysteine	0.405216183	0.015742345	positive
Family_XIII_AD3011_group	Luteoforol	0.403814051	0.01614216	positive
Parasutterella	5beta-Dihydrotestosterone	-0.401988936	0.016675301	negtive
Negativibacillus	Tamoxifen	-0.398720432	0.017666914	negtive
Iba-1	N-Stearoyl Taurine	-0.395726455	0.018618015	negtive
Butyricimonas	Aucubin	0.391621164	0.019991252	positive

Blautia	N-Stearoyl Taurine	-0.391596639	0.020666368	negtive
Family_XIII_AD3011_group	5-Hydroxymethyl-2-furancarboxaldehyde	-0.388811237	0.020978956	negtive
Butyricimonas	N-Stearoyl Taurine	-0.387277602	0.02153492	negtive
Blautia	Nandrolone	0.384313725	0.023317312	positive
Negativibacillus	3,7-Dihydroxy-12-oxocholanoic acid	-0.383757728	0.022857283	negtive
Clostridium_sensu_stricto_1	N-Stearoyl Taurine	0.381113432	0.02389435	positive
GFAP	S-Allylcysteine	0.380278478	0.02422978	positive
Clostridium_sensu_stricto_1	Asparagine	-0.380271502	0.024232599	negtive
Dorea	Hydrocortamate	-0.379661314	0.02448021	negtive
Blautia	Hydrocortamate	-0.375910364	0.026723352	negtive
Family_XIII_AD3011_group	Iba-1	0.373840209	0.026949157	positive
Akkermansia	Nicotinic acid mononucleotide	-0.372673113	0.027468028	negtive
Family_XIII_AD3011_group	Fructose-6-phosphate	0.371845439	0.027840955	positive

Dorea	2-Hexaprenyl-3-methyl-5-hydroxy-6-methoxy-1,4-benzoquinone	-0.369882159	0.028742221	negtive
Clostridium_sensu_stricto_1	5-Hydroxymethyl-2-furancarboxaldehyde	0.364555485	0.03130876	positive
Family_XIII_AD3011_group	Piperine	0.364273925	0.031449475	positive
Clostridium_sensu_stricto_1	Myricetin	0.362871626	0.032158028	positive
Clostridium_sensu_stricto_1	2-Hexaprenyl-3-methyl-5-hydroxy-6-methoxy-1,4-benzoquinone	0.362450661	0.03237326	positive
Akkermansia	Asparagine	-0.36224672	0.032477953	negtive
Family_XIII_AD3011_group	5-Hydroxy-7-methoxy-2-tritriacontyl-4H-1-benzopyran-4-one	0.361890301	0.032661586	positive
Clostridium_sensu_stricto_1	S-Allylcysteine	-0.360766802	0.033245986	negtive
Akkermansia	Octaprenyl diphosphate	-0.359006084	0.034178979	negtive
Butyricimonas	S-Allylcysteine	0.357853471	0.034801215	positive
GFAP	Hydrocortamate	-0.356396934	0.035600684	negtive

Akkermansia	S-Allylcysteine	-0.354215579	0.036825871	negtive
GFAP	5-Hydroxy-7-methoxy-2-tritriacontyl-4H-1-benzopyran-4-one	0.353306381	0.037346541	positive
Parasutterella	Momordicoside I	-0.353105961	0.037462116	negtive
Butyricimonas	L-glutamine	0.35238899	0.037877942	positive
Blautia	Senecioic acid	0.350980392	0.039339601	positive
Negativibacillus	Myricetin	-0.343002173	0.043675521	negtive
Parasutterella	5-Hydroxy-7-methoxy-2-tritriacontyl-4H-1-benzopyran-4-one	0.342320892	0.044122664	positive
Family_XIII_AD3011_group	3alpha-3-Hydroxytirucalla-7,24-dien-21-oic acid	0.341699598	0.044533641	positive
Blautia	Fasciculic acid B	0.341176471	0.045479106	positive
Iba-1	Nicotinic acid mononucleotide	0.340420446	0.045389477	positive
Dorea	3-Hydroxyvalproic acid	-0.33881896	0.046479554	negtive
Family_XIII_AD3011_group	2-Hexaprenyl-3-methyl-5-hydroxy-6-methoxy-1,4-benzoquinone	-0.33609107	0.048384633	negtive
Blautia	Soyasapogenol A	0.333613445	0.050728754	positive

Akkermansia	L-glutamine	-0.331249334	0.051919568	negtive
Blautia	2,4-Dihydroxyacetophenone 5-sulfate	0.331092437	0.052583574	positive
GFAP	Myricetin	-0.328300999	0.054171103	negtive
Parasutterella	Piperine	-0.324112334	0.057502664	negtive
Iba-1	S-Allylcysteine	0.312274895	0.067801485	positive
Butyricimonas	Amphibine H	0.306711531	0.073116303	positive
Akkermansia	2-Hexaprenyl-3-methyl-5-hydroxy-6-methoxy-1,4-benzoquinone	0.304901557	0.07491374	positive
Blautia	10-nitro-9E-octadecenoic acid	0.304761905	0.075402791	positive
Negativibacillus	Asparagine	0.30238912	0.077465585	positive
Clostridium_sensu_stricto_1	GFAP	-0.3023225	0.077534157	negtive
Blautia	Aucubin	0.301680672	0.078517136	positive
Blautia	Ecgonine methyl ester	0.299439776	0.080844841	positive
Clostridium_sensu_stricto_1	N2, N2-Dimethylguanosine	-0.29874466	0.081286599	negtive

Akkermansia	5-Hydroxymethyl-2-furancarboxaldehyde	0.298702079	0.081332089	positive
Negativibacillus	Fasciculic acid B	0.297686556	0.08242284	positive
Parasutterella	Luteoforol	-0.293437917	0.087109259	negtive
Family_XIII_AD3011_group	3,7-Dihydroxy-12-oxocholanoic acid	-0.29332604	0.087235376	negtive
Akkermansia	Luteoforol	-0.293207088	0.087369623	negtive
Clostridium_sensu_stricto_1	Fructose-6-phosphate	-0.292009224	0.088730392	negtive
Dorea	5-Hydroxy-p-mentha-6,8-dien-2-one	-0.288485074	0.092828329	negtive
Dorea	N2, N2-Dimethylguanosine	0.287622207	0.093853416	positive
Family_XIII_AD3011_group	Momordicoside I	0.2854741	0.096442964	positive
Akkermansia	N2, N2-Dimethylguanosine	-0.285175948	0.096806653	negtive
Clostridium_sensu_stricto_1	Glyuranolide	-0.284151215	0.098064608	negtive
Negativibacillus	3alpha-3-Hydroxytirucalla-7,24-dien-21-oic acid	0.282296346	0.10037325	positive
Family_XIII_AD3011_group	Myricetin	-0.2780428	0.105823158	negtive

Family_XIII_AD3011_ group	Fasciculic acid B	0.277762373	0.106190166	positive
Family_XIII_AD3011_ group	Nandrolone	0.277341734	0.106742483	positive
Family_XIII_AD3011_ group	Soyasapogenol A	0.277341734	0.106742483	positive
Family_XIII_AD3011_ group	5beta-Dihydrotestosterone	0.276079815	0.108412459	positive
Butyricimonas	Tamoxifen	-0.273924642	0.111310011	negtive
Butyricimonas	5-Hydroxy-7-methoxy-2-tritriacontyl-4H-1-benzopyran-4-one	0.270561885	0.115947002	positive
Akkermansia	Decanoylcarnitine	0.268972769	0.118187986	positive
Clostridium_sensu_stri cto_1	L-glutamine	-0.267593268	0.120159501	negtive
Clostridium_sensu_stri cto_1	Luteoforol	-0.267031981	0.120968654	negtive
Negativibacillus	10-nitro-9E-octadecenoic acid	0.265481117	0.123225517	positive
Akkermansia	Aucubin	-0.262209703	0.128088768	negtive

Family_XIII_AD3011_group	Aucubin	0.260375935	0.130876312	positive
Butyricimonas	2-Hexaprenyl-3-methyl-5-hydroxy-6-methoxy-1,4-benzoquinone	-0.256410279	0.1370576	negtive
Akkermansia	5-Hydroxy-p-mentha-6,8-dien-2-one	0.254742151	0.1397209	positive
Dorea	Octaprenyl diphosphate	0.251381809	0.145200929	positive
Family_XIII_AD3011_group	N2, N2-Dimethylguanosine	0.25014037	0.147264651	positive
Butyricimonas	3alpha-3-Hydroxytirucalla-7,24-dien-21-oic acid	0.249544649	0.148262515	positive
Butyricimonas	10-nitro-9E-octadecenoic acid	0.245341201	0.155443837	positive
Family_XIII_AD3011_group	3-Hydroxyvalproic acid	-0.244111202	0.157592042	negtive
Family_XIII_AD3011_group	Tamoxifen	-0.243690563	0.158331592	negtive
Family_XIII_AD3011_group	Asparagine	0.242148217	0.161064715	positive
Butyricimonas	Asparagine	0.238895916	0.166939037	positive
Akkermansia	Hydrocortamate	-0.238538972	0.167592978	negtive

Blautia	Glyuranolide	-0.237535014	0.168944919	negtive
Iba-1	Asparagine	0.233185895	0.177621088	positive
Blautia	Nicotinic acid mononucleotide	0.232212885	0.178913757	positive
Clostridium_sensu_stricto_1	Iba-1	-0.231372133	0.181113553	negtive
Butyricimonas	Myricetin	-0.231329711	0.181195816	negtive
Butyricimonas	5-Hydroxymethyl-2-furancarboxaldehyde	-0.231049481	0.181739889	negtive
Butyricimonas	Momordicoside I	0.229788447	0.184202518	positive
Family_XIII_AD3011_group	Ecgonine methyl ester	0.224341139	0.19511098	positive
Parasutterella	N2, N2-Dimethylguanosine	-0.223124869	0.197607033	negtive
Negativibacillus	Epothilone A	0.221020511	0.201978098	positive
Negativibacillus	Benazepril	0.218597978	0.207092748	positive
Iba-1	Myricetin	-0.217846569	0.208697235	negtive
Negativibacillus	Momordicoside I	0.215605437	0.213533686	positive
Akkermansia	Tamoxifen	0.212191194	0.221049061	positive

Parasutterella	GFAP	-0.210664024	0.224468463	negtive
Clostridium_sensu_stricto_1	Phenylbutyrylglutamine	0.209219487	0.227735861	positive
Family_XIII_AD3011_group	Epothilone A	0.207655767	0.231309102	positive
Butyricimonas	3,7-Dihydroxy-12-oxocholanoic acid	-0.207510177	0.231643711	negtive
Parasutterella	5-Hydroxy-p-mentha-6,8-dien-2-one	0.206737167	0.233425799	positive
Family_XIII_AD3011_group	Adrenoyl ethanolamide	0.206253635	0.234545229	positive
Akkermansia	10-nitro-9E-octadecenoic acid	0.205005437	0.237451689	positive
Dorea	5-Hydroxymethyl-2-furancarboxaldehyde	-0.204787012	0.23796278	negtive
Negativibacillus	2,4-Dihydroxyacetophenone 5-sulfate	0.201212741	0.246431509	positive
Clostridium_sensu_stricto_1	Aucubin	-0.200519549	0.248096955	negtive
Akkermansia	5beta-Dihydrotestosterone	0.200355829	0.248491397	positive
Negativibacillus	Hydrocortamate	-0.19964522	0.250208281	negtive
Family_XIII_AD3011_group	N-Stearoyl Taurine	-0.199102761	0.251524205	negtive

Family_XIII_AD3011_group	10-nitro-9E-octadecenoic acid	0.199102761	0.251524205	positive
Akkermansia	Phenylbutyrylglutamine	0.198242371	0.253620813	positive
Parasutterella	S-Allylcysteine	0.194691505	0.26239611	positive
Family_XIII_AD3011_group	Senecioic acid	0.19293338	0.266814118	positive
Akkermansia	GFAP	-0.192482476	0.267955014	negtive
Akkermansia	3-Hydroxyvalproic acid	0.191620202	0.270145664	positive
Dorea	Decanoylcarnitine	-0.190118279	0.273989266	negtive
Negativibacillus	Senecioic acid	0.18938508	0.27587849	positive
Family_XIII_AD3011_group	Amphibine H	0.188726984	0.277581398	positive
Negativibacillus	Soyasapogenol A	0.188530068	0.278092265	positive
Akkermansia	Senecioic acid	0.186125211	0.284380532	positive
Blautia	Asparagine	0.184593838	0.287224225	positive
Blautia	5beta-Dihydrotestosterone	0.183473389	0.290200044	positive

Negativibacillus	5beta-Dihydrotestosterone	0.182972493	0.292762231	positive
Parasutterella	Fructose-6-phosphate	-0.180684922	0.298941866	negtive
Butyricimonas	Epothilone A	0.18018777	0.300295772	positive
Parasutterella	2-Hexaprenyl-3-methyl-5-hydroxy-6-methoxy-1,4-benzoquinone	0.176202816	0.311288662	positive
Parasutterella	Octaprenyl diphosphate	-0.173821697	0.317976436	negtive
Parasutterella	Aucubin	-0.171160446	0.325556405	negtive
Butyricimonas	Fasciculic acid B	0.170940186	0.326188744	positive
Blautia	Phenylbutyrylglutamine	0.170308123	0.326661481	positive
Clostridium_sensu_stricto_1	5-Hydroxy-p-mentha-6,8-dien-2-one	0.168526227	0.333168761	positive
Butyricimonas	5beta-Dihydrotestosterone	0.164915245	0.343780157	positive
Akkermansia	Fructose-6-phosphate	-0.162595377	0.350704735	negtive
Negativibacillus	Phenylbutyrylglutamine	0.15632463	0.369840271	positive
Family_XIII_AD3011_group	Glyuranolide	-0.155496452	0.372412903	negtive

Family_XIII_AD3011_group	2,4-Dihydroxyacetophenone 5-sulfate	0.154374747	0.375914156	positive
Blautia	S-Allylcysteine	0.153501401	0.377195476	positive
Parasutterella	Iba-1	-0.150729984	0.387423859	negtive
Dorea	Tamoxifen	-0.149275926	0.392072144	negtive
Butyricimonas	Phenylbutyrylglutamine	0.148802031	0.393594016	positive
Akkermansia	Benazepril	0.14582861	0.403220473	positive
Clostridium_sensu_stricto_1	Piperine	-0.1449522	0.406083296	negtive
Clostridium_sensu_stricto_1	Benazepril	0.140882874	0.419526646	positive
Parasutterella	Nicotinic acid mononucleotide	0.138244975	0.42837275	positive
Butyricimonas	Soyasapogenol A	0.137592838	0.430575511	positive
Clostridium_sensu_stricto_1	Hydrocortamate	-0.135410332	0.437992905	negtive
Clostridium_sensu_stricto_1	3-Hydroxyvalproic acid	0.134568403	0.440872877	positive

Clostridium_sensu_stricto_1	Fasciculic acid B	0.132744222	0.447148178	positive
Negativibacillus	Ecgonine methyl ester	0.13067428	0.454327194	positive
Family_XIII_AD3011_group	Benazepril	0.130258074	0.455778132	positive
Clostridium_sensu_stricto_1	10-nitro-9E-octadecenoic acid	0.130218433	0.455916453	positive
Clostridium_sensu_stricto_1	Amphibine H	-0.130218433	0.455916453	negtive
Akkermansia	Ecgonine methyl ester	0.127511973	0.465413366	positive
Clostridium_sensu_stricto_1	Adrenoyl ethanolamide	0.127131358	0.466757288	positive
Negativibacillus	Glyuranolide	-0.123406681	0.480016579	negtive
Dorea	Amphibine H	0.11993846	0.492536735	positive
Dorea	S-Allylcysteine	-0.118212727	0.498828176	negtive
Parasutterella	Decanoylcarnitine	0.111912599	0.522137293	positive
Negativibacillus	Nandrolone	0.111151514	0.524988827	positive
Dorea	Fructose-6-phosphate	0.111022172	0.525474184	positive

Akkermansia	Iba-1	-0.110993214	0.525582879	negtive
Butyricimonas	Ecgonine methyl ester	0.110550661	0.527245382	positive
Parasutterella	L-glutamine	0.110091743	0.52897206	positive
Family_XIII_AD3011_group	Hydrocortamate	-0.105440336	0.546626303	negtive
Parasutterella	Tamoxifen	0.104349044	0.550808172	positive
Clostridium_sensu_stricto_1	Soyasapogenol A	0.101733151	0.560893046	positive
Butyricimonas	Nandrolone	0.101443192	0.562016128	positive
Family_XIII_AD3011_group	Phenylbutyrylglutamine	0.101093726	0.563371075	positive
Butyricimonas	Senecioic acid	0.097800205	0.576213853	positive
Negativibacillus	Adrenoyl ethanolamide	0.093908779	0.591555564	positive
Blautia	Myricetin	-0.093837535	0.590553736	negtive
Clostridium_sensu_stricto_1	Epothilone A	0.093313856	0.593916691	positive
Dorea	Myricetin	0.093189595	0.594410373	positive

Akkermansia	Fasciculic acid B	0.091724082	0.600246245	positive
Parasutterella	Myricetin	-0.09104279	0.602967626	negtive
Butyricimonas	Hydrocortamate	-0.08939331	0.609578196	negtive
Akkermansia	Adrenoyl ethanolamide	0.085383708	0.625773799	positive
Dorea	L-glutamine	0.083122818	0.634983156	positive
Parasutterella	Asparagine	0.082358709	0.638107937	positive
Parasutterella	3-Hydroxyvalproic acid	0.07997759	0.647884583	positive
Clostridium_sensu_stricto_1	3alpha-3-Hydroxytirucalla-7,24-dien-21-oic acid	0.077036552	0.660040469	positive
Dorea	5-Hydroxy-7-methoxy-2-tritriacontyl-4H-1-benzopyran-4-one	-0.076795129	0.661042179	negtive
Butyricimonas	Benazepril	0.076782969	0.661092651	positive
Clostridium_sensu_stricto_1	3,7-Dihydroxy-12-oxocholanoic acid	0.068336613	0.696493032	positive
Parasutterella	5-Hydroxymethyl-2-furancarboxaldehyde	0.062889558	0.719667624	positive
Butyricimonas	Glyuranolide	-0.062351133	0.721972163	negtive
Parasutterella	N-Stearoyl Taurine	0.056726662	0.746186256	positive

Akkermansia	2,4-Dihydroxyacetophenone 5-sulfate	-0.049173125	0.779081153	negtive
Parasutterella	Amphibine H	0.046781988	0.789576484	positive
Clostridium_sensu_stricto_1	Momordicoside I	0.044622265	0.799087527	positive
Clostridium_sensu_stricto_1	2,4-Dihydroxyacetophenone 5-sulfate	-0.042798084	0.807143166	negtive
Dorea	Nicotinic acid mononucleotide	0.040554731	0.817076613	positive
Akkermansia	Momordicoside I	0.039451218	0.821973314	positive
Akkermansia	3,7-Dihydroxy-12-oxocholanoic acid	-0.038042246	0.828235053	negtive
Akkermansia	Nandrolone	-0.037478657	0.83074269	negtive
Clostridium_sensu_stricto_1	Nandrolone	0.031572357	0.857116636	positive
Dorea	Asparagine	-0.023585021	0.893021435	negtive
Akkermansia	Soyasapogenol A	0.020289198	0.907902193	positive
Butyricimonas	2,4-Dihydroxyacetophenone 5-sulfate	0.010928963	0.950315787	positive
Clostridium_sensu_stricto_1	Ecgonine methyl ester	-0.008980582	0.959165152	negtive

Dorea	N-Stearoyl Taurine	-0.008916288	0.959457253	negtive
Akkermansia	Glyuranolide	-0.008876524	0.959637916	negtive
Clostridium_sensu_stricto_1	Decanoylcarnitine	0.008699938	0.960440227	positive
Dorea	Aucubin	0.008341044	0.962070969	positive
Clostridium_sensu_stricto_1	Senecioic acid	-0.007156401	0.967454782	negtive
Akkermansia	3alpha-3-Hydroxytirucalla-7,24-dien-21-oic acid	0.006763066	0.969242685	positive
Clostridium_sensu_stricto_1	5beta-Dihydrotestosterone	0.005893507	0.973195774	positive
Akkermansia	Epothilone A	-0.005494991	0.975007675	negtive
Akkermansia	Amphibine H	-0.001549869	0.99294987	negtive
Butyricimonas	Adrenoyl ethanolamide	0.001401149	0.993626362	positive
