# Supplementary material for: Metabolomics combined with intestinal microbiota reveals the mechanism of compound Qilian tablets against diabetic retinopathy
Source: Front Microbiol. 2024 Aug 16;15:1453436. doi: 10.3389/fmicb.2024.1453436 (PMC11362098; doi:10.3389/fmicb.2024.1453436)
Supplement: Supplementary file 3 [file Data_Sheet_3.PDF]

Immunohistochemical analysis of GFAP and Iba-1 in the retina

After the retinal sections were prepared, deparaffinized, and rehydrated, they were washed 3 times with phosphate buffered saline (PBS) (pH 7.4), 3 minutes each time. Each section was spiked with 50 μl of hydrogen peroxide blocking solution, incubated for 10 minutes at room temperature to block endogenous catalase activity, and rinsed three times with PBS for 3 minutes each time. Antigen retrieval was performed on the tissue sections using citrate buffer. 100 μl of 5% Bovine Serum Albumin (BSA) solution was added to each section and incubated for 30 minutes at room temperature. Sections were sequentially subjected to primary antibody incubation, washing, secondary antibody incubation, and washing. Subsequently, 100 μl of freshly prepared DAB solution was added, and the sections were dried by gradient alcohol dehydration and sealed with neutral resin. In addition, the control group was replaced with PBS for primary antibody, following the same steps as before. Finally, the stained positive substances were observed under the microscope, and the average optical density values were measured.
